# GC-MS Determination of Undeclared Phthalate Esters in Commercial Fragrances: Occurrence, Profiles and Assessment of Carcinogenic and Non-Carcinogenic Risk Associated with Their Consumption among Adult Consumers

**DOI:** 10.3390/molecules28041689

**Published:** 2023-02-10

**Authors:** Ahmed Mostafa, Heba Shaaban

**Affiliations:** Department of Pharmaceutical Chemistry, College of Clinical Pharmacy, Imam Abdulrahman Bin Faisal University, King Faisal Road, P.O. Box 1982, Dammam 31441, Saudi Arabia

**Keywords:** risk assessment, phthalate esters, gas chromatography (GC-MS), Saudi Arabia, cosmetics, systemic exposure, fragrances

## Abstract

Phthalates are chemicals that are extensively used in the manufacturing of cosmetic products. The occurrence of phthalate esters in personal care products may pose adverse effects on consumers’ health. In this work, a simple, fast and reliable GC-MS method was developed and validated for concurrent determination of phthalate esters in fragrances. Simple procedures were employed for sample preparation and clean up. The recoveries achieved were in the range of 94.9% to 105.6% with RSD ≤ 4.06. The detection limits were in the range of 0.0010 to 0.0021 µg/mL. The GC-MS method was utilized to investigate the occurrence of phthalate esters in different brands of perfumes sold in the Saudi Arabian market. Diethyl phthalate was detected in all analyzed samples, with a maximum concentration of 5766 µg/mL, and di (2-ethylhexyl) phthalate was detected in the majority of the analyzed samples (95%), with a mean concentration of 55.9 µg/mL and a highest concentration of 377.7 µg/mL. Additionally, the exposure to phthalate esters due to the consumption of perfumes was investigated among the adult Saudi population for the first time. It was found that the systemic exposure dose, measured at mean concentrations, ranged from 4.59 × 10^−4^ to 4.29 × 10^−2^ (mg/kg/day) and from 5.00 × 10^−4^ to 4.68 × 10^−2^ (mg/kg/day) for male and female users, respectively. Moreover, the non-carcinogenic risk of the investigated phthalate esters and the carcinogenic risk of DEHP were also evaluated. The non-carcinogenic risk values of the detected phthalate esters were greater than 100, which indicates that exposure to these phthalate esters is unlikely to produce non-carcinogenic health effects to consumers. However, at maximum DEHP concentrations, the carcinogenic risk values were 5.49 × 10^−5^ for male users and 5.98 × 10^−5^ for female users, which indicates the possibility of DEHP to pose a carcinogenic health effect if present at high levels. Regular monitoring of undeclared chemicals such as phthalate esters in personal care products marketed in Saudi Arabia is extremely important to ensure consumers’ safety. To the best of the authors’ knowledge, this is the first study to assess the health risk associated with consumption of perfumes in Saudi Arabia.

## 1. Introduction

Phthalate esters (Phthalates) are substances extensively used in the manufacturing of different products, including cosmetic products, pharmaceutical formulations, toys, food products etc. [1]. Low molecular weight phthalate esters such as dimethyl phthalate are commonly used in fragrances as stabilizing diluent, and diethyl phthalate is utilized as a fixative or carrier for fragrances [2]. The primary role of phthalates in perfumes is to act as fixatives, which help to make the fragrance last longer on the skin. Phthalates help to keep the fragrance molecules suspended in the product, preventing them from evaporating too quickly. They also help to ensure that the fragrance is released more slowly, providing a longer-lasting scent. Phthalates also help to make the fragrance more stable, preventing it from breaking down or changing over time [3]. Phthalate esters of high molecular weight such as di(2-ethylhexyl) phthalate are widely utilized as plasticizers in the manufacturing of polyvinyl chloride plastics [2]. These chemicals can be leached from the plastic containers into products, causing adverse effects on health [1]. Phthalates can interfere with the endocrine system, causing serious reproductive and developmental effects [4,5,6]. However, their degree of toxicity depends on their chemical structures.

Human exposure to phthalate esters due to daily use of personal care products may lead to observed urinary concentrations of phthalate metabolites such as monoethyl phthalate (the primary metabolite of diethyl phthalate) [7]. Recent studies determined the urinary levels of phthalate esters in different population ages and indicated an association between the consumption of personal care products and the occurrence of phthalate ester metabolites in the consumers’ urine samples [8,9,10,11,12].

Because of the concerns about the adverse effects of phthalate on human health, governmental regulations have been developed to restrict the use of phthalates in different products. For instance, the European Union prohibited the use of dibutyl phthalate, bis(2-ethylhexyl) phthalate and benzyl butyl phthalate in cosmetics [13]. Regular monitoring of undeclared substances including phthalates in personal care products is highly required to ensure the consumers’ safety. In this regard, various analytical methodologies were developed to investigate the occurrence of these chemicals in different care products e.g., [14,15,16,17].

Saudi Arabia is the largest market for fragrances in the Arabian Gulf region. Its perfume market was estimated at USD 1.6 billion in 2021 and expected to reach USD 2.1-billion by 2027 with a growth rate of 4.59% during 2022–2027, according to IMARC Group [18]. The use of perfumes by both males and females is very popular in Saudi Arabia [19]. Various brands of fragrances, either locally manufactured or imported from other countries, are available in the Saudi market.

The main objective of this work was to investigate the occurrence pattern of phthalate esters contained in perfumes commercially available in the Saudi market and to evaluate the health risk attributed to the usage of perfumes among adult consumers. The levels of five phthalate esters in commercial perfumes were determined using gas chromatography-mass spectrometry (GC-MS). To the best of our knowledge, this is the first study to investigate the exposure to phthalate esters from the consumption of perfumes available in the Saudi market, and also to assess their health risk among the adult Saudi population.

## 2. Materials and Methods

### 2.1. Reagents and Standards

Dimethyl phthalate (DMP), dibutyl phthalate (DBP), diethyl phthalate (DEP), di (2-ethylhexyl) phthalate (DEHP) and benzyl butyl phthalate (BBP) were obtained from Sigma-Aldrich, Germany (Steinheim, Germany) with minimum purity ≥98%. Ethanol was purchased from Sigma-Aldrich, Germany. A stock standard solution of each analyte (1000 µg/mL) was prepared in ethanol and kept at −20 °C. All working solutions were freshly prepared from the stock solutions using proper dilution. Glassware used in all procedures was washed with deionized water and then with acetone.

### 2.2. Instrumentation

**Figure 1:**
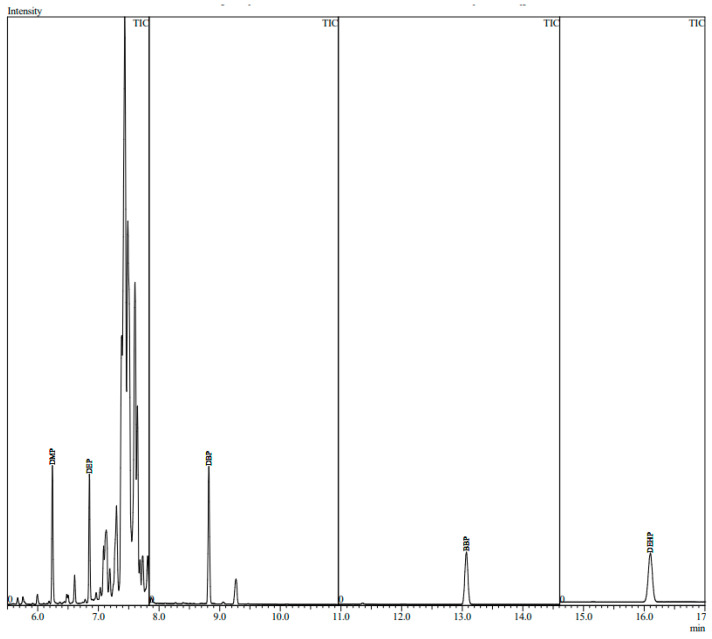
The GC-MS ion chromatogram of the investigated phthalate esters in a spiked perfume sample.

GC-MS analysis was conducted using a 2010 plus gas chromatograph (Shimadzu, Japan) equipped with a split/splitless injector and coupled to a QP2010 Ultra mass spectrometer. Electron ionization mode was utilized for the MS at 70 eV. The injection was done in the splitless mode and the injector temperature was kept at 280 °C. Chromatographic separation was achieved on Rxi-5MS capillary column (30 m × 0.25 mm i.d. × 1.00 µm) (Restek, Bellefonte, PA, USA). Helium was used as carrier gas with a flow rate of 1 mL/min. The temperature gradient started at 100 °C for 0.5 min and then increased to 280 °C at 30 °C/min, and held for 15 min. The ion source and transfer line temperatures were 250 °C and 280 °C, respectively. A solvent delay time of 5 min was applied. Selective ion monitoring mode was utilized to enhance method sensitivity and selectivity. Table 1 shows the MS parameters and retention times of target analytes. Positive identification of the target analytes was performed using the ratio of the monitored ions and retention times (Table 1). Data acquisition and processing was performed utilizing GC-MS Solution^®^ version 4.52 (Shimadzu, Japan). Figure 1 represents the total ion chromatogram of GC-MS analysis of the target phthalate esters in a spiked perfume sample. All investigated analytes were separated under the optimized conditions.

### 2.3. Sampling and Preparation

Forty perfumes of different brands, including store brands, national brands and international brands were collected from pharmacies, local markets and fragrance shops located in Al Khobar and Dammam cities, Saudi Arabia. No pretreatment of the samples was required as all samples were clear liquids. Samples were diluted in ethanol (1:5), then 1 µL was used for GC-MS analysis. In the event of an excessive phthalate concentration, an appropriate extra dilution of the sample in ethanol was performed to ensure accurate results within the linearity range (Supplementary Materials).

### 2.4. Statistical Analysis

Data analysis was performed using Microsoft Excel (Office 365) and Statistical Package for Social Science (SPSS version 22) (IBM Corp., Armonk, NY, USA). Descriptive statistical parameters such as percentage, mean, standard deviation and frequency were used to present the concentrations of phthalate esters in the analyzed perfumes samples. For determination of the association between the concentrations of the investigated phthalate esters in the analyzed samples, Pearson’s correlation test was applied.

### 2.5. Health Risk Assessment

The health risk assessment of the investigated phthalate esters based on systemic exposure was performed. The non-cancer risk assessment was expressed as the margin of safety (*MOS*) according to the following equation:

(1) $$MOS=\frac{NOAEL}{\;SED},$$

where *NOAEL* is the no observed adverse effect level and *SED* is the systemic exposure dose. The *NOAEL* values of the investigated phthalates were identified in various studies such as ref. [20,21,22,23,24]. *MOS* values greater than 100 are known to be safe and values below 100 indicate a possibility of causing health risk to consumers.

Additionally, *SED* (systemic exposure dose) (mg/kg/day) was calculated using the following equation [25]:

(2) $$SED=\frac{A\times 1000\times C\left(\%\right)/100\times D\left(\%\right)/100}{BW},$$

where *A* is the cosmetic usage per day (g/day), *C* (%) is concentration of phthalate esters in perfumes determined by GC-MS, *D* (%) is the dermal absorption rate and *BW* is the body weight (kg). The consumption use of perfumes per day is 0.75 [26]. *SED* values were calculated using the average detected concentrations, and the maximum concentrations as well, in order to represent the worst-case exposure scenario. The dermal absorption rates of the investigated phthalate esters were 5%, except for DBP, which was 10% [26]. The average body weight of Saudi males was 67.4 kg and the average body weight for Saudi females was 61.9 kg [27].

The carcinogenic risk for DEHP was expressed as lifetime cancer risk (*LCR*) according to the following equation:

(3) $$LCR=\frac{SED}{human\;T25\;\left(HT25\right)/0.25},$$

where *human T*25 (*HT*25) is a toxicity value that relates to a chronic dose which causes tumors at a specific tissue region in 25% of experimental animals, after correcting the rate using spontaneous carcinogenesis factor [28]. The HT25 value for DEHP determined in other studies was 95.73 [26,29]. *LCR* values of ≤ 10^−5^ indicate safety [25,30,31]. As a result, any value greater than 10^−5^ indicates that there is some carcinogenic risk to human health [25].

## 3. Results and Discussion

### 3.1. Method Validation

To evaluate the performance of the GC-MS method, different parameters were monitored, including linearity, precision, accuracy, limit of detection (LODs) and limit of quantification (LOQs). The results of method validation are summarized in Table 2. Linearity was studied in the range of 0.007–10 µg/mL, and determination coefficients (*r^2^*) higher than 0.9990 were obtained for all investigated analytes. LODs and LOQs were calculated based on signal-to-noise (S/N) ratios of 3 and 10, respectively. LODs and LOQs were 0.0010–0.0021 µg/mL and 0.0031–0.0065 µg/ mL, respectively, for the studied analytes.

Method accuracy was determined by spiking phthalate esters into real perfume samples at three different concentration levels (0.05, 0.1 and 5 µg/mL). These samples were analyzed in five replicates. The intra-day precision was evaluated by calculating the %RSD of the five replicates taken on the same day (Table 2). The inter-day precision was calculated as the %RSD for three replicates analyzed on three successive days (*n* = 3 × 3), at the same concentration levels. The obtained recovery was in the range of 94.9–105.6%, indicating the good accuracy of the method. The obtained %RSD values were satisfactory (≤4.06% for intra-day precision and ≤3.94% for inter-day precision). Procedural blanks (non-spiked ethanol) were used to monitor for potential contamination. An instrumental blank and a quality control sample (5 µg/mL) were also analyzed after every ten samples in each sequence to ensure proper performance of the GC-MS, and to detect any possible cross-contamination. When necessary, the sample concentration was adjusted correspondingly.

### 3.2. Occurrence and Distribution of Phthalate Esters in the Analyzed Perfume Samples

The descriptive data such as the mean, maximum concentrations and frequency for each phthalate ester in the analyzed samples were illustrated in Table 3. The concentrations of phthalate esters varied widely among the analyzed samples.

In all analyzed samples (*n* = 40), at least one phthalate ester was found at a detectable level.

DEHP was found in most of the analyzed samples (95%, 38 out of 40) at levels ranging from ˂LOD to 377.67 µg/mL, with an overall average concentration of 55.92 mg/L. The high detection frequency of DEHP in this study may be attributed to the vast use of DEHP as a plasticizer during the manufacturing of the plastic spray bottles [32]. The high DEHP detection frequency in this study reflected this manufacturing practice. It was found that DEHP concentrations in the presented study were larger than that determined in other previous studies e.g., [33]. For example, Al-Saleh and Elkhatib [33] detected DEHP at concentrations up to 147.54 µg/mL in 97.9% of the analyzed samples. Additionally, the mean value of DEHP in this study was higher than values reported by Al-Saleh and Elkhatib (8.46 µg/mL) [33], Koo and Lee (0.678 µg/mL) [34] and Guo and Kannan (2.71 µg/mL) [35]. On the other hand, DEHP concentrations found in the presented study were lower than that determined in another previous study [26]. Kim et al. [26] found DEHP in 93.3% of the analyzed samples, with a maximum concentration of 600 µg/mL.

Even though the usage of DEHP in cosmetic products was banned by different regularity authorities due to its reproductive and developmental toxicity [36], our study found DEHP in 38 out of 40 samples. DEP was found in all the analyzed samples. It was found that DEP had the highest levels in all analyzed perfume samples, as shown in Table 3. DEP was found to have a mean value of 771.67 µg/mL and a maximum value of 5766 µg/mL. The high levels of DEP in perfumes were also reported in other studies: mean = 1621.63 µg/mL, *n* = 47 [33]; mean = 3044.24 µg/mL, *n* = 42 [34]; mean =15,235.91 µg/mL, *n* = 11 [37]; mean value = 3420 µg/mL, *n* = 12 [35], median value = 1679 µg/mL, *n* = 30 [38] and median value = 4686 µg/mL, *n* = 70 [39]. The DEP levels found in the presented study were much smaller than that reported by Al-Saleh and Elkhatib [33] and Koniecki et al. [38].

DMP was the least-detected phthalate ester in the analyzed perfume samples (found in 8 out of the 40 analyzed samples) with a maximum concentration of 60.00 µg/mL. The DMP detection rate reported in this study was greater than those documented in other studies. For example, Peters [40] detected DMP in only one sample out of 36 analyzed perfume products. Also, Hubinger et al. [37] and Koniecki et al. [38] did not detect DMP in any of the analyzed samples (11 and 30 perfume samples, respectively). The DMP concentrations found here were below the values reported by Al-Saleh and Elkhatib [33], who detected DMP in 72.3% of the samples at levels ranging from 0.15 to 405.24 µg/mL.

Although the EU banned the use of DBP in cosmetic products, it was detected in 27.5% of the analyzed samples (11 out of 40), with a maximum concentration of 66.00 µg/mL. According to the Directive 76/768/ EEC, the European Union (EU) banned the use of DBP in cosmetic products [13]. The DBP levels in this study were higher than those reported by Guo and Kannan [35], who reported a mean value of 0.21 µg/mL in 12 perfume samples, and Al-Saleh and Elkhatib, who found DBP with a mean value of 0.03 µg/mL and a maximum value of 0.59 µg/mL [33]. The DBP levels reported in this study were also higher than those reported by Hubinger [37], who did not detect DBP in any of the analyzed samples and Sanchez-Prado et al., who reported a higher median value of 0.9 µg/mL [39]. On the other hand, this study reported DBP levels lower than that reported by Koo and Lee, who detected DBP at very high levels of 444.7 µg/mL [34]. BBP was detected in 57.5% of the analyzed samples (23 out of 40). The levels of BBP were generally low, with a mean value of 4.13 µg/mL and a maximum value of 22.50 µg/mL.

**Figure 2:**
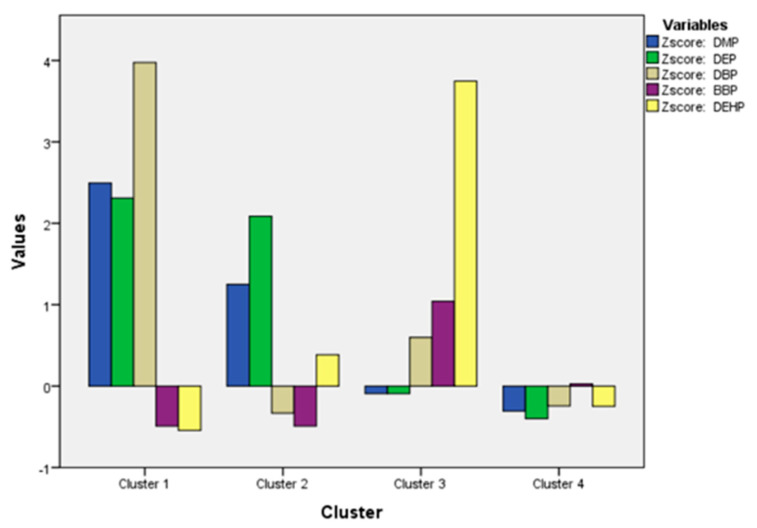
Cluster loading for the studied phthalate esters in the analyzed samples (*n* = 40).

K-mean cluster analysis was performed to investigate the pattern of phthalate ester distribution in the analyzed samples. It classified the analyzed samples into various clusters according to the distribution and amount of the investigated phthalate esters in the samples. As shown in Figure 2, the samples were classified into four clusters with frequent distribution and varied amounts of phthalate esters. Cluster 1 represents the largest amounts of DMP, DEP and DBP and cluster 3 represents the highest level of DEHP. On the other hand, cluster 4 represents the samples containing a lower abundance of most of phthalate esters. The pattern of significant (high amount) and non-significant (low amount) distribution of phthalate esters in the analyzed samples (*n* = 40) is given in Table 4. The greatest amount of DBP was found in samples of cluster 1, with the highest F-ratio of 95.99 (*p*-value of 0.00). BBP was non-significantly distributed (low amount or sparse distribution), with low F-ratio of 1.238 and *p*-value of 0.310. DMP, DEP and DEHP were found to be significantly distributed in the analyzed samples (Table 4). The varied distribution of phthalate esters in the analyzed samples reflects an urgent need for a proper and continual monitoring of personal care products including perfumes, to ensure the proper quality and safety of commercial local and imported cosmetic products.

### 3.3. Correlations among the Phthalate Esters Concentrations in the Analyzed Perfume Samples

Phthalate esters have endocrine effects even at low concentrations, and co-administration of phthalate esters can cause cumulative health effects. A mixture of phthalate esters was found in all the analyzed perfume samples. Therefore, the correlations among the detected phthalate ester concentrations were investigated using Pearson’s correlation analysis. The correlation data in terms of linear correlation coefficient values that are significant at the 0.01 and 0.05 levels were investigated, and any value ≥0.50 was considered to correlate the two bivariate data points.

Significant positive correlations were observed between different phthalate esters, including DMP-DEP (*p* < 0.01), DMP-DBP (*p* < 0.01) and DEP-DBP (*p* = 0.003). The pair observed with the highest positive correlation was DMP-DEP (correlation coefficient (r) = 0.612), revealing widespread sequestration of these phthalate esters in the 40 perfume samples. The next significantly high positive correlation is seen for the DMP-DBP pair, with a value of 0.599. Likewise, a high significant positive correlation value of 0.459 was observed for the DEP-DBP pair. Occurrence of multiple phthalate esters may cause cumulative health effects. The results of the Pearson’s correlation are illustrated in Table 5.

### 3.4. Health Risk Assessment

The detection of multiple phthalate esters in commercial perfumes necessitates the assessment of health risk associated with their usage in order to ensure consumer safety. The exposure to phthalate esters due to perfume consumption was investigated among the adult Saudi population for the first time. The non-carcinogenic risk of DMP, DEP, DBP, BBP and DEHP and carcinogenic risk of DEHP were also evaluated.

#### 3.4.1. Systemic Exposure Dosage (SED) of Phthalate Esters in Perfumes

The computed values of the systemic exposure dose for the investigated phthalate esters in different perfume samples at mean and maximum concentrations are presented in Table 6. It was noted that, at mean concentrations, SED values (mg/kg/day) ranged from 4.59 × 10^−4^ to 4.29 × 10^−2^ and from 5.00 × 10^−4^ to 4.68 × 10^−2^ for male and female users, respectively. Likewise, SED values (mg/kg/day) at maximum levels ranged from 2.50 × 10^−3^ to 3.21 × 10^−1^ for male users and from 2.73 × 10^−3^ to 3.49 × 10^−1^ for female users.

Among the detected phthalate esters, it was noted that DEP has the highest SED values for male and female users, ranging from 4.29 × 10^−2^ to 3.49 × 10^−1^ at mean and maximum concentrations, respectively. The values of systemic exposure to DBP reported in this study were higher than those reported by Kim et al. (6.25 × 10^−7^ mg/kg/day) [26]. Also, the estimation of mean systemic daily exposure of DEHP (3.11 × 10^−3^ mg/kg/day, calculated for male) was higher than the mean exposure reported by Kim et al. (3.75 × 10^−4^ mg/kg/day) [26]. Table 6 shows the mean and maximum SED values of the detected phthalate esters in the analyzed perfume samples for Saudi adults.

**Table 6:** Non observed adverse effect level (NOAEL) and systemic exposure dose (SED) (mg/kg/day) of phthalate esters for adult Saudi population.

		Systemic Exposure Dose (SED)			
	NOAEL *	Male		Female	
		Mean	Maximum	Mean	Maximum
DMP	3.75	1.15× 10^−3^	3.34× 10^−3^	1.26× 10^−3^	3.63× 10^−3^
DEP	0.15	4.29× 10^−2^	3.21× 10^−1^	4.68× 10^−2^	3.49× 10^−1^
DBP	0.66	8.45× 10^−4^	3.67× 10^−1^	9.20× 10^−4^	4.00× 10^−3^
BBP	50	4.59× 10^−4^	2.50× 10^−3^	5.00× 10^−4^	2.73× 10^−3^
DEHP	4.8	3.11× 10^−3^	2.10× 10^−2^	3.39× 10^−3^	2.29× 10^−2^

* Values are obtained from ref. [20,21,22,23,24].

#### 3.4.2. Non-Carcinogenic Risk Assessment

**Figure 3:**
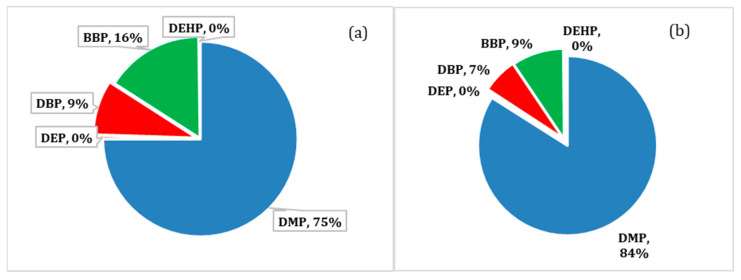
(**a**) The relative contribution of each phthalate ester to the non-carcinogenic health risk at mean concentration and (**b**) The relative contribution of each phthalate ester to the non-carcinogenic health risk at maximum concentration (calculated for male users).

To assess the non-carcinogenic health risk of phthalate esters, the margin of safety (MOS) value for each detected phthalate ester was determined. At mean concentrations, the non-carcinogenic risks for the investigated phthalate esters ranged from 1.54 × 10^3^ to 5.2 × 10^5^ and from 1.42 × 10^3^ to 4.77 × 10^5^ for male and female adults, respectively, whereas, at maximum concentrations, MOS values ranged from 2.28 × 10^2^ to 1.8 × 10^5^ for male and from 2.1 × 10^2^ to 1.65 × 10^5^ for female users. The contributions of each phthalate ester relative to the non-carcinogenic risk (MOS), calculated at mean and maximum concentrations for adult male users, are illustrated in Figure 3a,b, respectively. It was found that DMP contributed the majority of the total non-carcinogenic risk (75%), followed by BBP (16%) at mean levels. The values of MOS were below the values documented by other researchers such as Kim et al. [26]. Generally, all MOS values of the detected phthalate esters were higher than 100 (Table 7), which indicated that exposure to these phthalate esters is unlikely to produce non-carcinogenic risk to the consumers of perfumes.

Though all the values reported in this study are considered safe, it is worthy to mention that co-existence of phthalate esters may cause serious health effects due to possible synergistic actions. Additionally, rates of consumption vary greatly among individuals, therefore, periodic investigation of phthalate esters in perfumes and other cosmetic products is highly required. Also, continual monitoring of undeclared chemicals in cosmetics from the Saudi markets, where exposure to chemicals such as phthalate esters is still uncharacterized, is of paramount importance, and should receive strong consideration in order to protect consumers from the health risks related to the usage of cosmetic products.

#### 3.4.3. Carcinogenic Risk Assessment

The carcinogenic health risk due to the consumption of perfumes containing phthalate esters was evaluated by calculating LCR caused by DEHP (classified by IARC in Group 2B as “possibly carcinogenic to humans”) [41]. The calculated values of LCR of DEHP in the analyzed perfume samples at mean and maximum concentrations are presented in Table 7. Apparently, the carcinogenic risk values calculated for maximum levels were higher than those calculated for average levels. It was observed that, at average levels, the carcinogenic risk values were 8.12 × 10^−6^ and 8.85 × 10^−6^ for males and females, respectively. According to the Scientific Committee on Consumer Safety (SCCS) Notes of Guidance for the testing of cosmetic ingredients [25], LCR values ≤ 10^−5^ indicate safety. Therefore, DEHP at average concentrations is unlikely to cause carcinogenic risk to consumers. However, at maximum DEHP concentrations, LCR values were 5.49 × 10^−5^ for male users and 5.98 × 10^−4^ for female users, which indicates the possibility of DEHP to pose a carcinogenic health effect if present at high concentrations. The values of carcinogenic risk calculated at maximum concentrations were greater than those reported for perfume samples collected from other markets, such as the Korean market [26].

Minimizing the levels of phthalate esters, especially DEHP, in perfumes and other cosmetic products should receive close attention.

Humans are exposed to phthalates from different sources on a daily basis. Exposure to high doses of phthalate esters, especially DEHP, may cause serious health problems such as reproductive toxicity [42]. Prolonged exposure to even low doses of phthalate esters is regarded as a hidden threat which can pose serious consequences, including carcinogenicity and mutagenicity [43]. Additionally, exposure to two or more phthalate esters simultaneously may have cumulative adverse health effects [43].

The method of perfume application can have an impact on the potential health risks associated with perfume use. For example, spraying perfume around the head or in the air can lead to exposure to chemicals contained in the perfume including phthalates. Inhalation exposure can cause irritation of the eyes, nose, and throat. When a significant quantity of droplets are inhaled, phthalates are adsorbed directly through the respiratory system, leading to respiratory problems [44]. Also, applying perfume directly to the skin can lead to dermal exposure to phthalates, causing skin irritation, allergic reactions and other skin problems. Additionally, phthalates can be absorbed through the skin and may accumulate in the body over time, leading to potential health risks [45]. Furthermore, applying perfume to the hair can also lead to dermal exposure to phthalates, causing scalp irritation and other hair-related problems. Additionally, if the perfume is applied to the hair and then exposed to heat, such as from a hair dryer or straightener, the fragrance, and any chemicals it contains, may be released into the air, leading to potential inhalation exposure [46]. The application of perfumes to clothing is the least likely source of phthalate exposure, e.g., DEHP transport from clothing to the body via evaporation and inhalation is negligible [47]. The health risk posed by harmful chemicals greatly depends on exposure; therefore, reducing consumption can minimize the exposure to harmful ingredients. Thus, consumers should be aware of the health risk due to the usage of products containing harmful chemicals such as phthalate esters. Raising public awareness regarding the undeclared chemicals in cosmetics and their adverse health effects may greatly affect the consumers’ choices.

## 4. Conclusions

The levels of phthalate esters in different fragrances available in Saudi Arabia were determined using GC-MS. It was found that DEHP was found in 95% of the analyzed samples, with a mean concentration of 55.92 µg/mL. The results showed that DMP contributed the majority of the total non-carcinogenic risk. No significant non-carcinogenic risk was found due to exposure to phthalate esters contained in the analyzed perfumes. However, at high concentrations, DEHP may have carcinogenic effects on consumers’ health. Minimizing the consumption of personal care products can significantly reduce the exposure to such undeclared harmful substances.

## Figures and Tables

**Table 1:** Retention times and GC-MS parameters of the investigated analytes.

Analyte	Retention Time (Min)	Selected Ions (*m/z*)	
		Quantifier Ion (*m/z*)	Qualifier Ions (*m/z*)
DMP	6.25	163	77, 133
DEP	6.86	149	177, 105
DBP	8.83	149	223, 104
BBP	13.10	149	91, 206
DEHP	16.13	149	167, 71

**Table 2:** Method performance for the investigated phthalate esters.

Analyte	Linearity Range (µg/mL)	r^2^	Recovery (%)			Inter-Day Precision (%RSD) (*n* = 3 × 3)			Intra-Day Precision (%RSD) (*n* = 5)			LOD (µg/mL)	LOQ (µg/mL)
			Low	Med	High	Low	Med	High	Low	Med	High		
DMP	0.010–10	0.9990	97.5	102.1	103.1	3.01	2.25	2.22	2.94	1.86	2.09	1.9 × 10^−3^	5.8 × 10^−3^
DEP	0.010–10	0.9996	94.9	96.9	97.5	2.99	2.46	2.67	2.75	2.04	2.25	1.5 × 10^−3^	4.6 × 10^−3^
DBP	0.007–7	0.9999	97.8	97.4	96.6	3.37	2.46	3.01	3.65	2.22	3.11	1.2 × 10^−3^	3.6 × 10^−3^
BBP	0.012–10	0.9997	98.2	105.6	101.9	3.21	3.06	2.99	3.20	2.68	2.94	2.1 × 10^−3^	6.5 × 10^−3^
DEHP	0.010–10	0.9999	100.3	98.8	97.9	3.94	1.58	2.55	4.06	1.77	2.29	1.0 × 10^−3^	3.1 × 10^−3^

**Table 3:** Concentrations (µg/mL) of the detected phthalate esters in the analyzed fragrance samples.

Sample Number	DMP	DEP	DBP	BBP	DEHP
1	5.70	0.90	19.30	0.40	85.70
2	27.00	2502.00	nd	nd	114.00
3	7.00	4.17	25.50	8.83	377.67
4	nd	1270.50	nd	6.00	309.00
5	nd	576.00	nd	22.50	96.00
6	nd	5766.00	nd	nd	186.00
7	nd	1.15	nd	1.80	48.30
8	nd	32.48	nd	0.98	37.57
9	nd	0.75	nd	0.10	49.60
10	nd	39.80	5.00	1.20	92.00
11	nd	13.05	3.10	0.10	46.05
12	20.60	1.15	nd	1.70	44.85
13	nd	1.05	nd	1.80	44.75
14	nd	0.60	3.65	nd	43.85
15	nd	874.00	2.00	nd	57.00
16	nd	26.30	nd	0.10	0.15
17	nd	12.35	nd	2.05	nd
18	12.00	3544.00	52.00	nd	16.00
19	nd	43.20	2.85	3.15	24.65
20	nd	468.50	nd	15.00	6.00
21	nd	0.35	nd	1.57	8.21
22	10.50	3440.50	nd	nd	31.50
23	60.00	4668.00	66.00	nd	6.00
24	nd	6.20	nd	0.10	44.75
25	44.00	3420.00	nd	nd	nd
26	nd	4.80	nd	0.10	33.50
27	nd	2.80	nd	nd	46.70
28	nd	2.45	0.95	nd	35.10
29	nd	3.25	nd	nd	17.15
30	nd	1602.00	nd	12.00	12.00
31	nd	6.35	nd	nd	42.10
32	nd	1.78	nd	nd	1.52
33	nd	4.84	nd	4.07	8.77
34	nd	11.00	nd	0.10	46.35
35	nd	693.00	nd	nd	6.00
36	nd	4.75	nd	nd	3.00
37	nd	1170.00	nd	10.00	10.00
38	nd	83.70	nd	nd	72.30
38	nd	20.90	1.80	1.30	14.70
40	nd	543.00	nd	nd	6.00
Mean	20.76	771.69	15.18	4.13	55.92
Maximum	60.00	5766.00	66.00	22.50	377.67
Frequency (%)	20.0	100.0	27.5	57.5	95.0
95 th percentile	53.60	3600.20	58.30	14.70	204.45

nd: not detected.

**Table 4:** K-mean cluster analysis for the analyzed samples (*n* = 40).

K-Mean Cluster Analysis				
Factors	F-Value	Significance	Clusters	Samples
Zscore: DMP	15.081	0.000	1	2
Zscore: DEP	68.732	0.000	2	4
Zscore: DBP	95.990	0.000	3	2
Zscore: BBP	1.238	0.310	4	32
Zscore: DEHP	48.246	0.000	Total	40

**Table 5:** Pearson’s correlation for the detected phthalate esters in the analyzed fragrance samples (*n* = 40).

	DMP	DEP	DBP	BBP	DEHP
DMP	1				
DEP	0.612 **	1			
	0.000				
DBP	0.599 **	0.459 **	1		
	0.000	0.003			
DBP	−0.145	−0.053	0.07	1	
	0.370	0.746	0.666		
DEHP	−0.05	0.107	0.093	0.221	1
	0.758	0.511	0.569	0.170	

** Correlation is significant at the 0.01 level.

**Table 7:** Estimated carcinogenic (MOS) and non-carcinogenic (LCR) health risks of investigated phthalate esters in the analyzed perfume samples.

	Margin of Safety (MOS)				LCR			
	Male		Female		Male		Female	
	Mean	Maximum	Mean	Maximum	Mean	Maximum	Mean	Maximum
DMP	5.20 × 10^−5^	1.80 × 10^−5^	4.77 × 10^−5^	1.65 × 10^−5^	-	-	-	-
DEP	3.49 × 10^−3^	4.68 × 10^−2^	3.21 × 10^−3^	4.29 × 10^−2^	-	-	-	-
DBP	5.92 × 10^−4^	1.36 × 10^−4^	5.44 × 10^−4^	1.25 × 10^−4^	-	-	-	-
BBP	1.09 × 10^−5^	2.00 × 10^−4^	1.00 × 10^−5^	1.83 × 10^−4^	-	-	-	-
DEHP	1.54 × 10^−3^	2.28 × 10^−2^	1.42 × 10^−3^	2.10 × 10^−2^	8.12 × 10^−6^	5.49 × 10^−5^	8.85 × 10^−6^	5.98 × 10^−5^

## Data Availability

Not available.

## Supplementary Materials

The following supporting information can be downloaded at: https://www.mdpi.com/article/10.3390/molecules28041689/s1.
