# Supplementary material for: GC-MS Determination of Undeclared Phthalate Esters in Commercial Fragrances: Occurrence, Profiles and Assessment of Carcinogenic and Non-Carcinogenic Risk Associated with Their Consumption among Adult Consumers
Source: Molecules. 2023 Feb 10;28(4):1689. doi: 10.3390/molecules28041689 (PMC9962674; doi:10.3390/molecules28041689)
Supplement: Supplementary file 1 [file molecules-28-01689-s001.zip › molecules-2141929-supplementary.pdf]

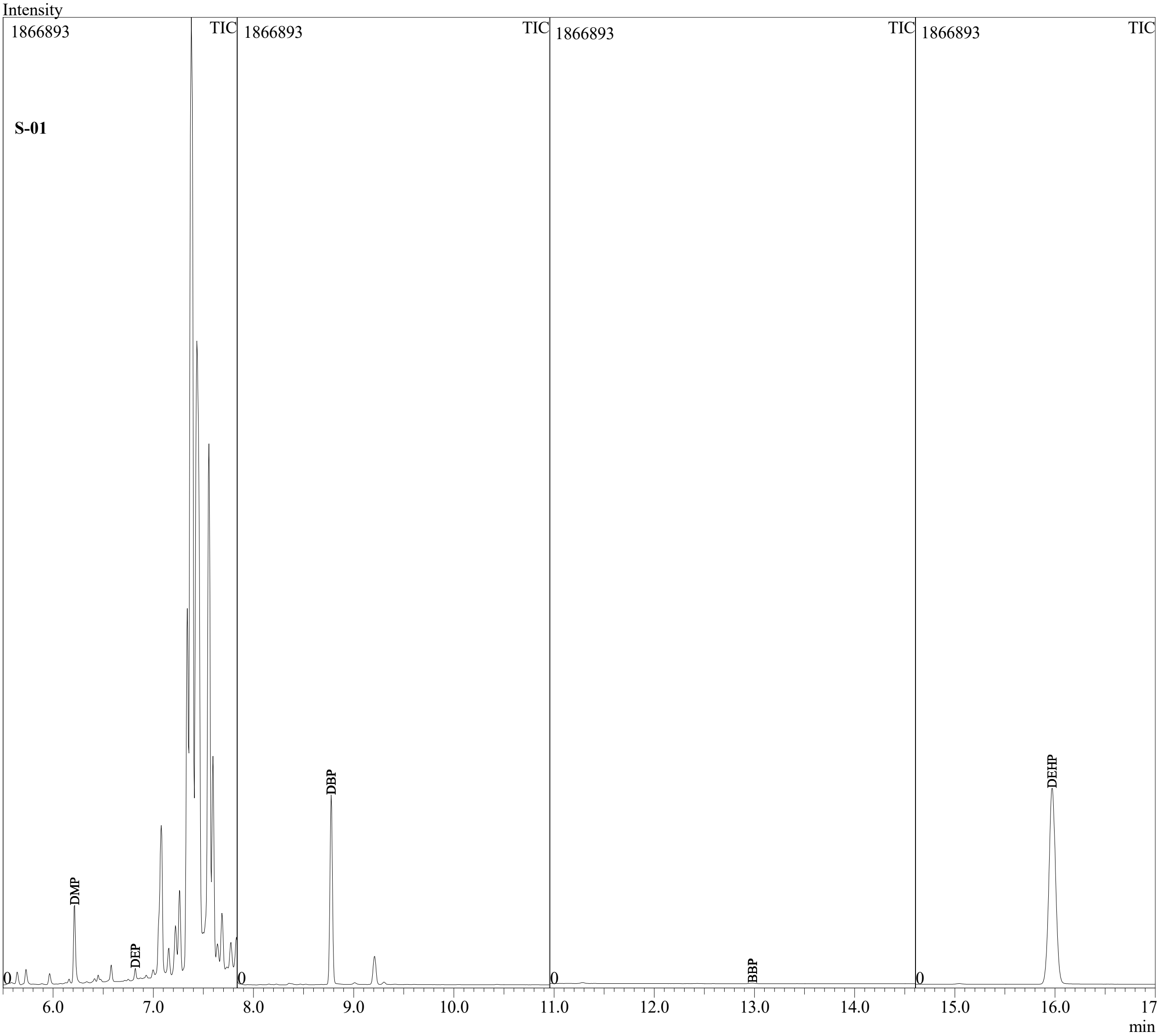
Intensity
1866893
TIC
S-01
DMP
DEP
DBP
BBP
DEHP
0
6.0
7.0
8.0
9.0
10.0
11.0
12.0
13.0
14.0
15.0
16.0
17
min

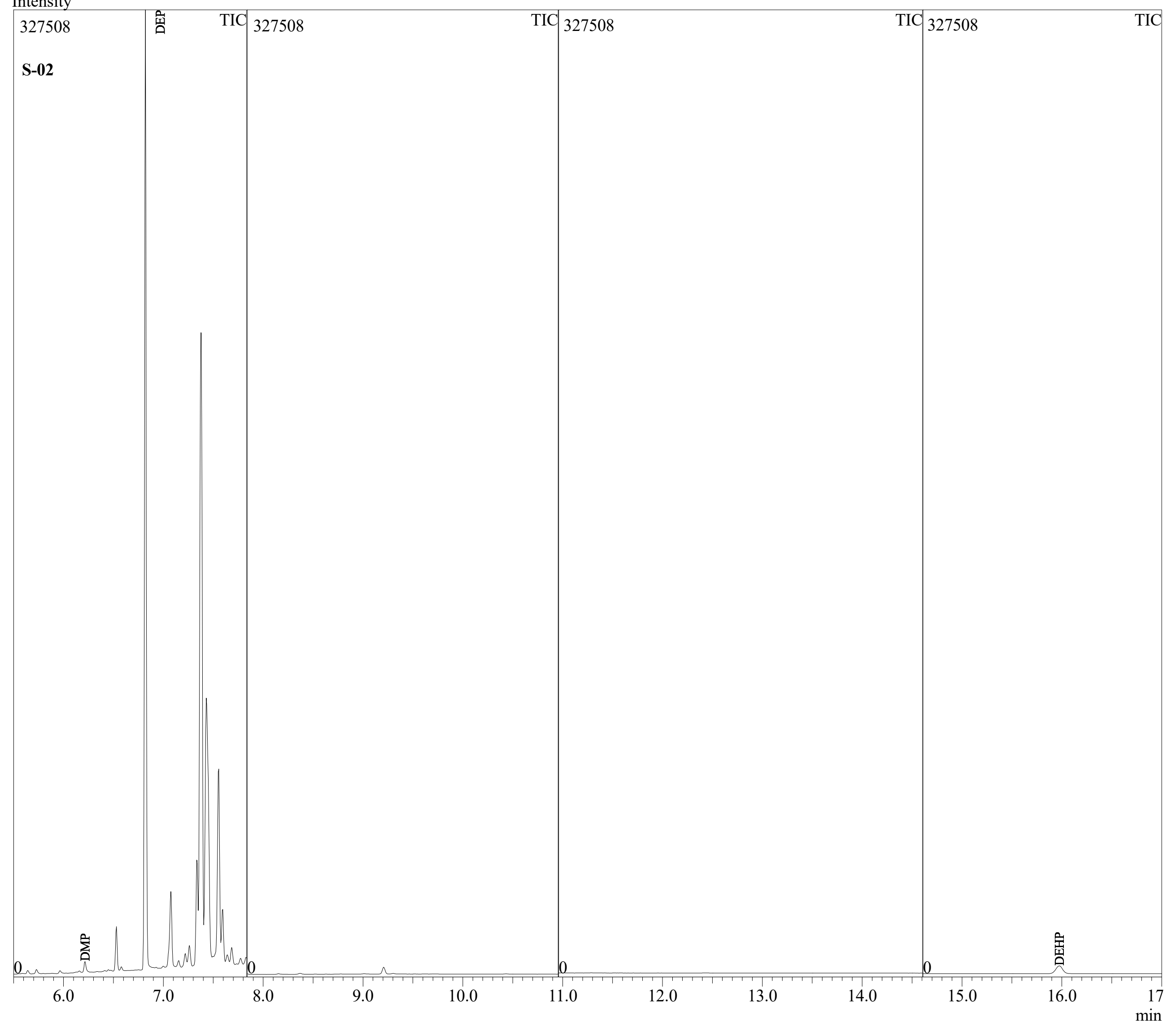
Intensity
327508
S-02
DEP
TIC
327508
TIC
327508
TIC
327508
TIC
DMP
DEHP
0
0
0
0
6.0
7.0
8.0
9.0
10.0
11.0
12.0
13.0
14.0
15.0
16.0
17
min

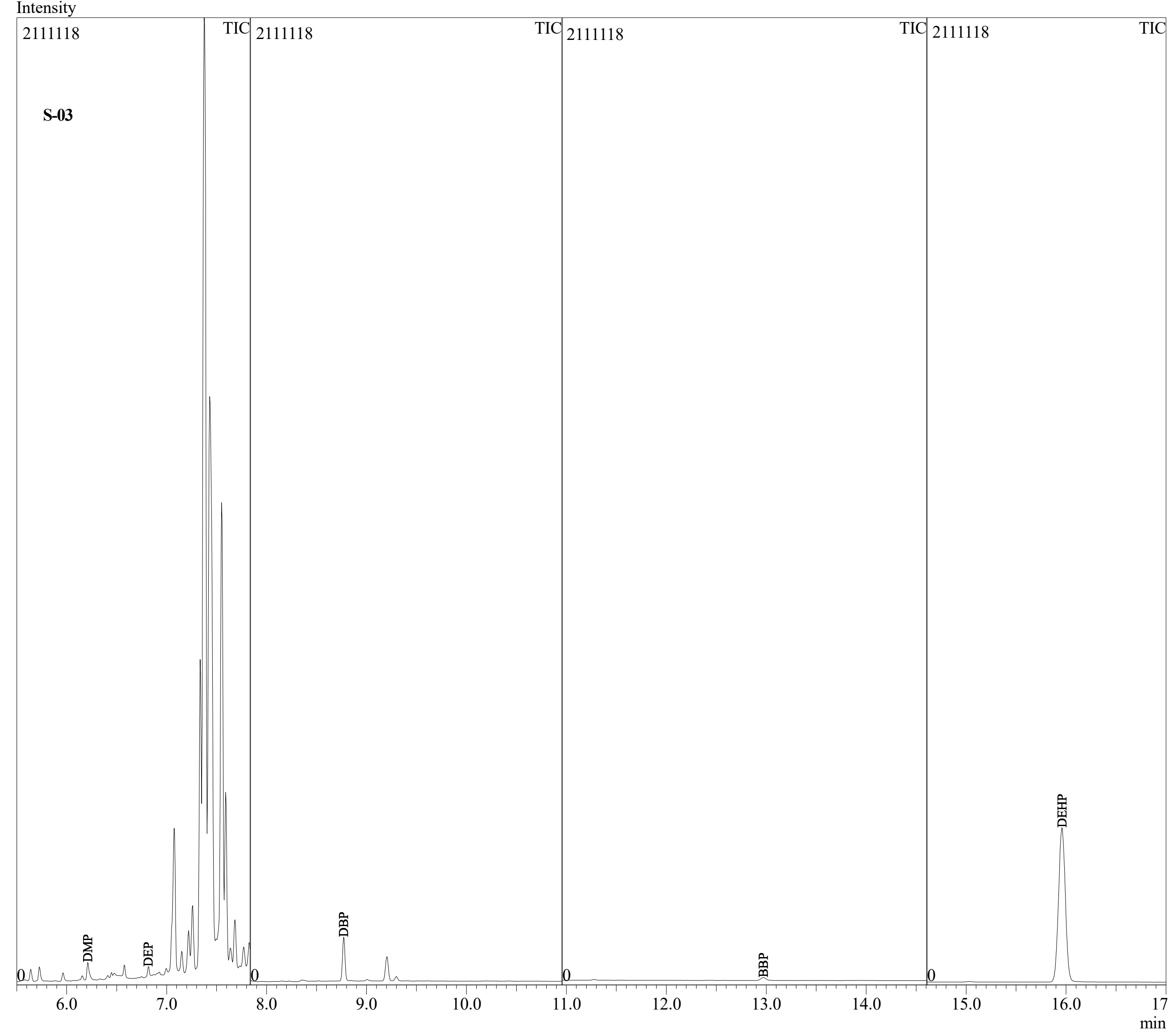

Intensity
2111118
TIC
S-03
DMP
DEP
DBP
BBP
DEHP
0
6.0
7.0
8.0
9.0
10.0
11.0
12.0
13.0
14.0
15.0
16.0
17
min

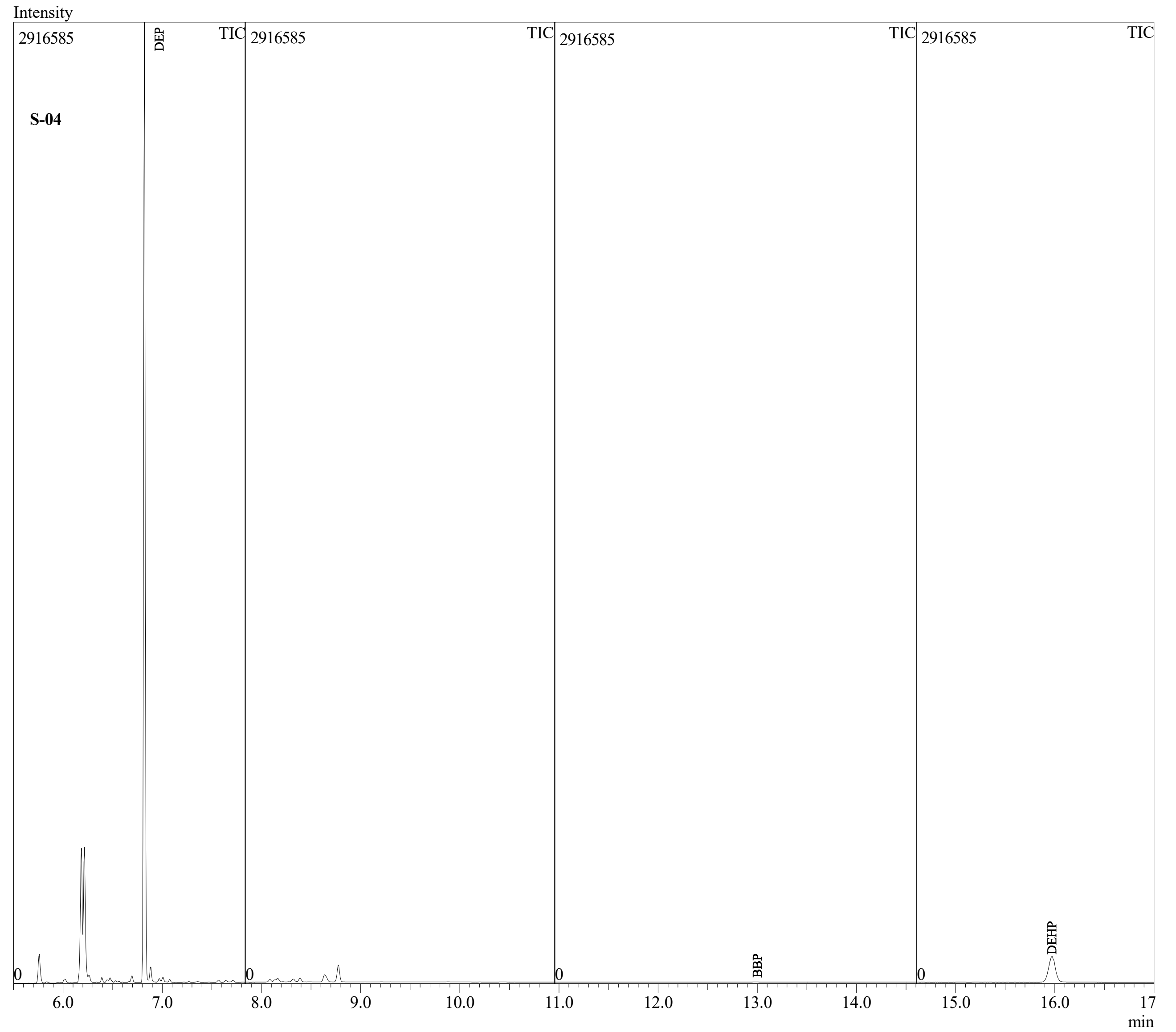

Intensity
2916585
TIC
S-04
DEP
BBP
DEHP
0
6.0
7.0
8.0
9.0
10.0
11.0
12.0
13.0
14.0
15.0
16.0
17
min

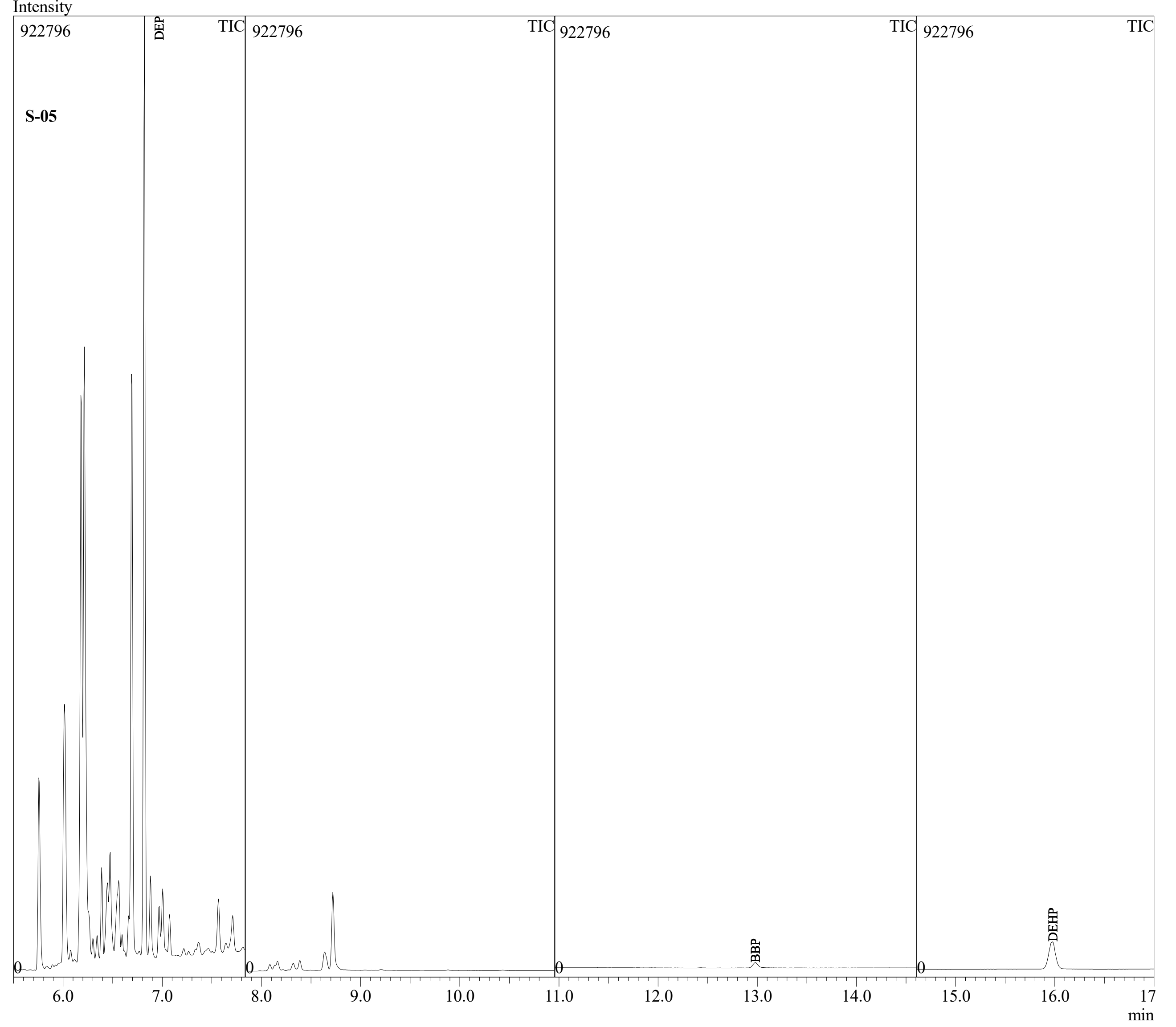

Intensity
922796
DEP
TIC
S-05
BBP
DEHP
0
6.0
7.0
8.0
9.0
10.0
11.0
12.0
13.0
14.0
15.0
16.0
17
min

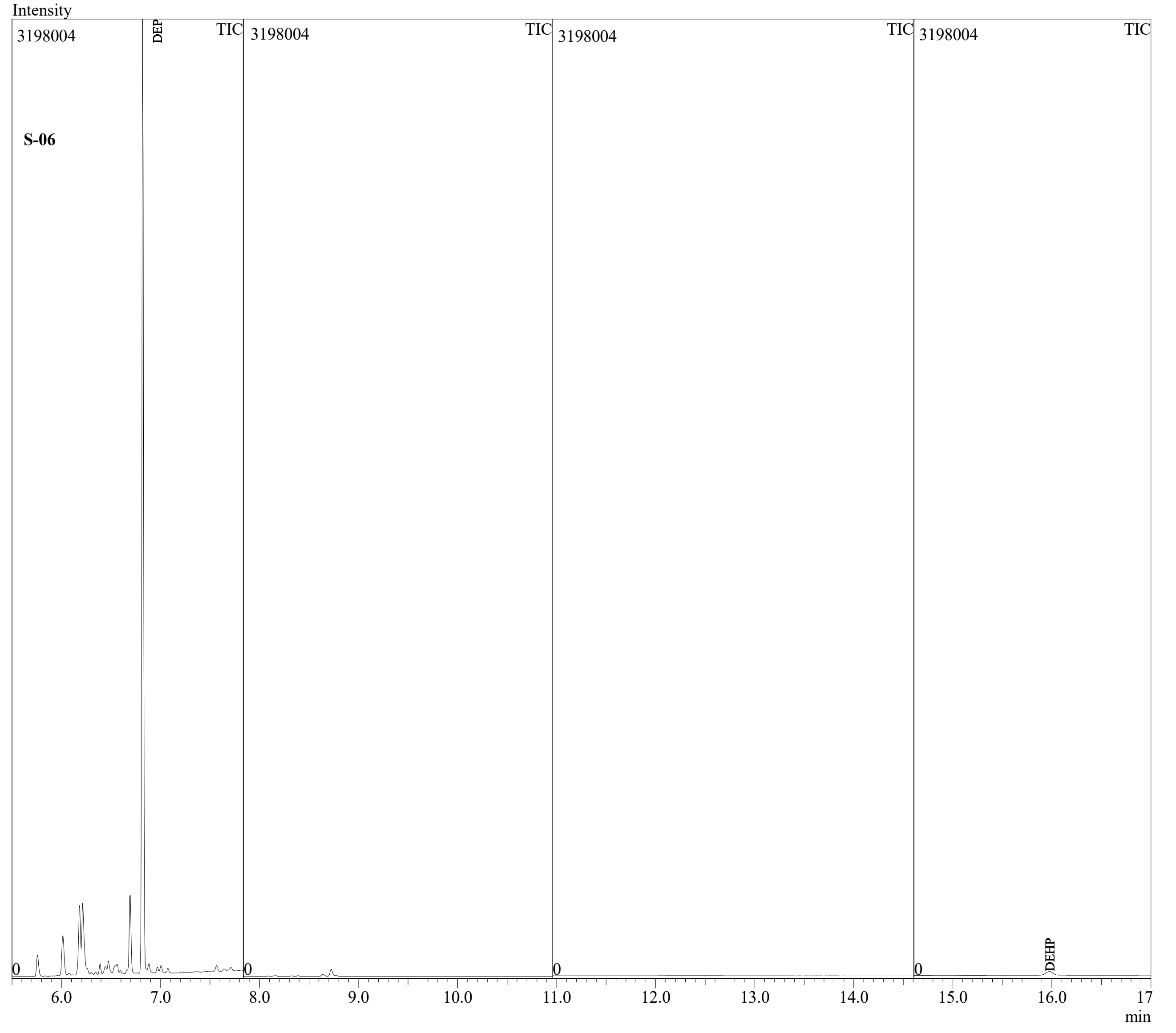

Intensity
3198004
TIC
S-06
DEP
DEHP
0
5.5
6.0
7.0
8.0
9.0
10.0
11.0
12.0
13.0
14.0
15.0
16.0
17
min

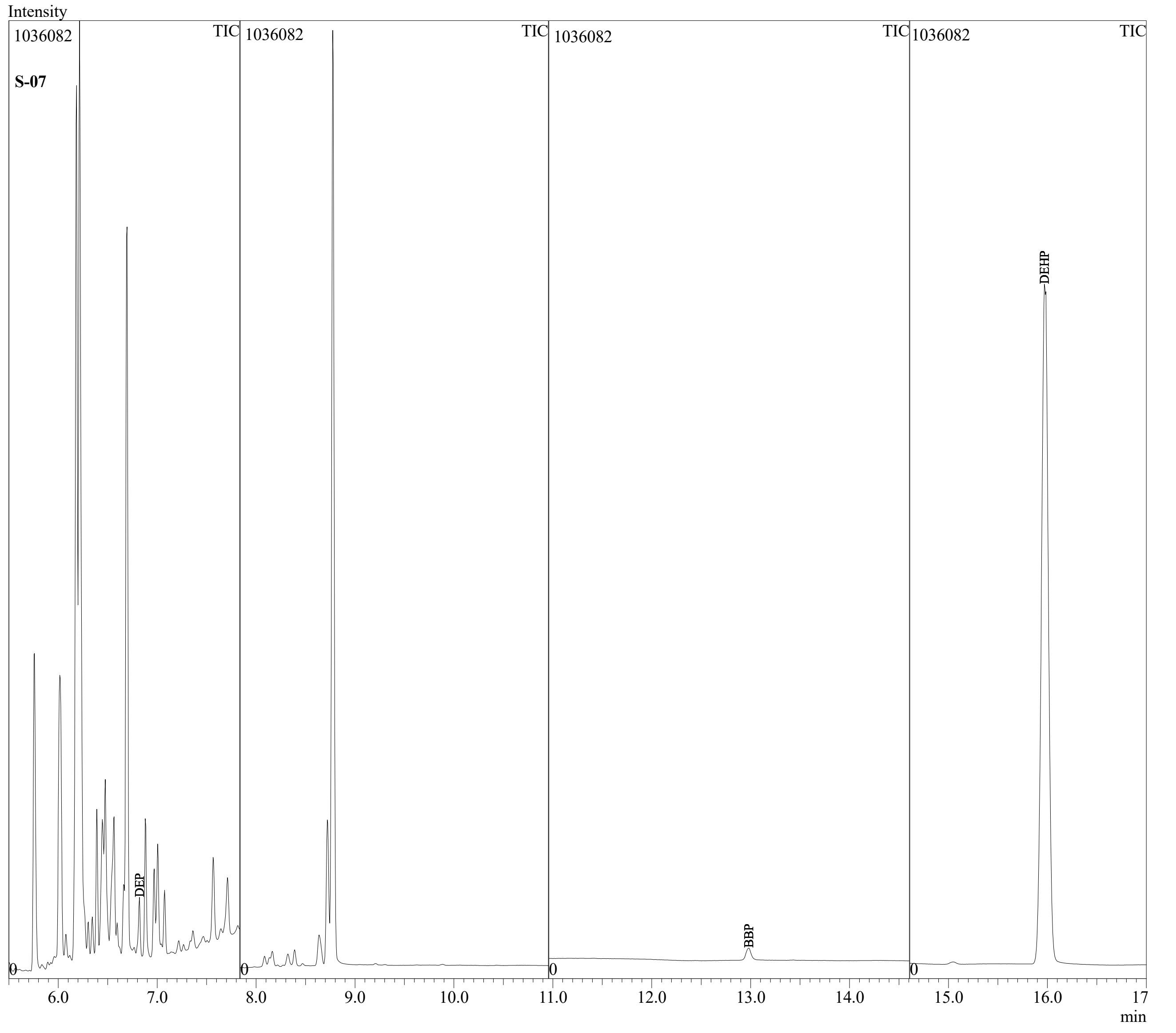
Intensity
1036082
S-07
TIC
DEP
1036082
TIC
1036082
TIC
BBP
1036082
TIC
DEHP
0
6.0
7.0
8.0
9.0
10.0
11.0
12.0
13.0
14.0
15.0
16.0
17
min

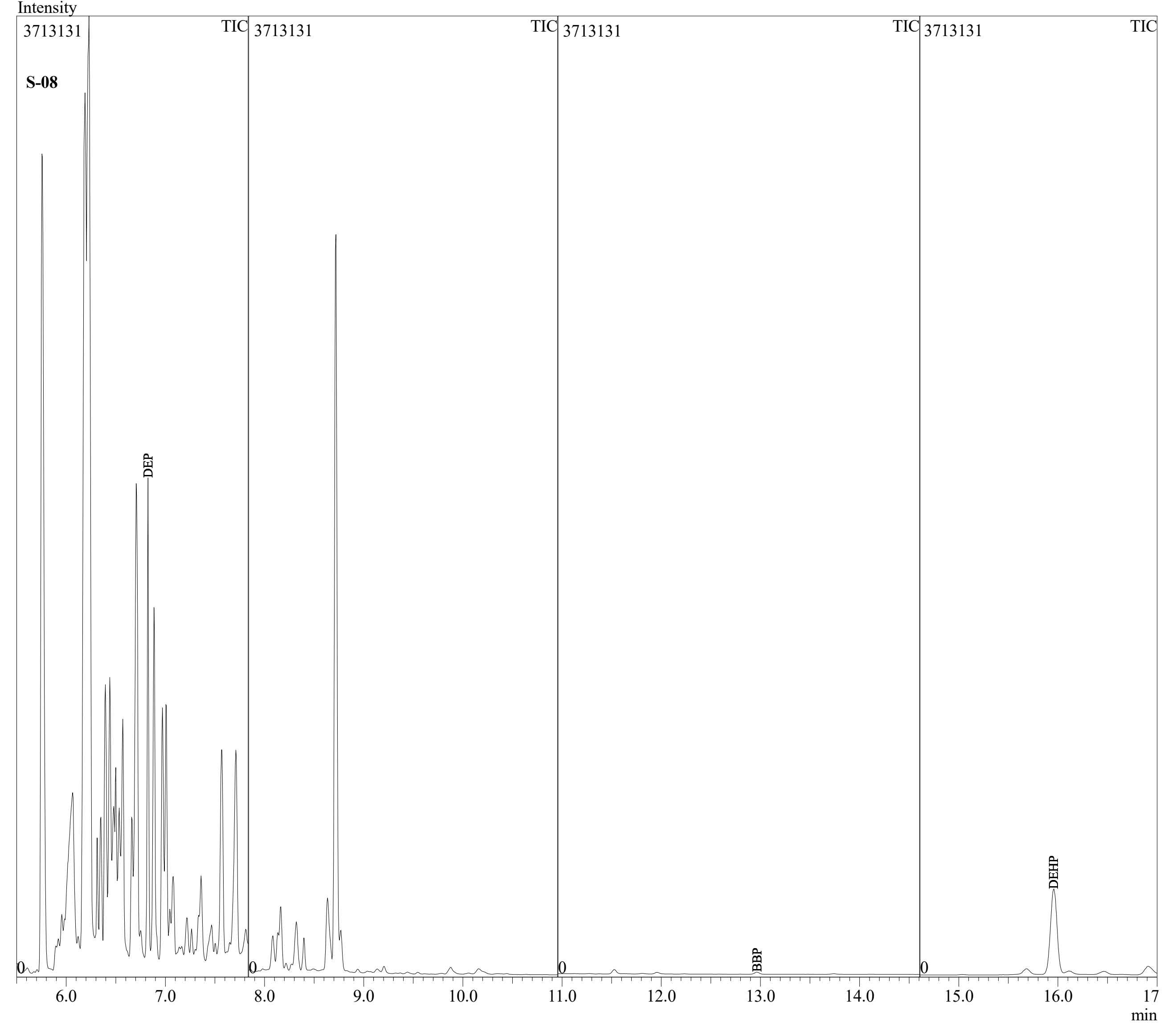

Intensity
3713131
TIC
S-08
DEP
BBP
DEHP
0
6.0
7.0
8.0
9.0
10.0
11.0
12.0
13.0
14.0
15.0
16.0
17
min

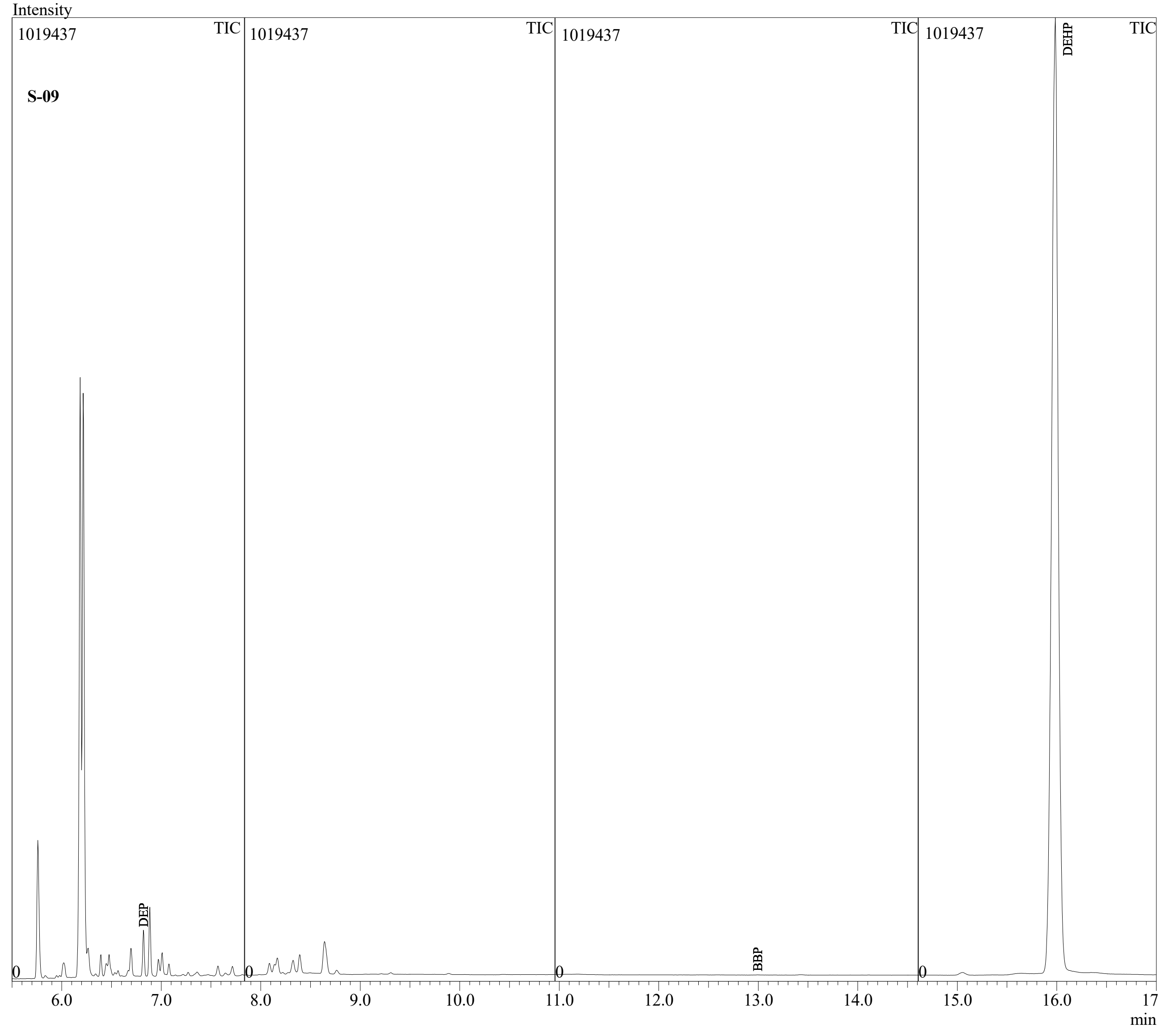

Intensity
1019437
TIC
S-09
DEP
BBP
DEHP
0
6.0
7.0
8.0
9.0
10.0
11.0
12.0
13.0
14.0
15.0
16.0
17
min

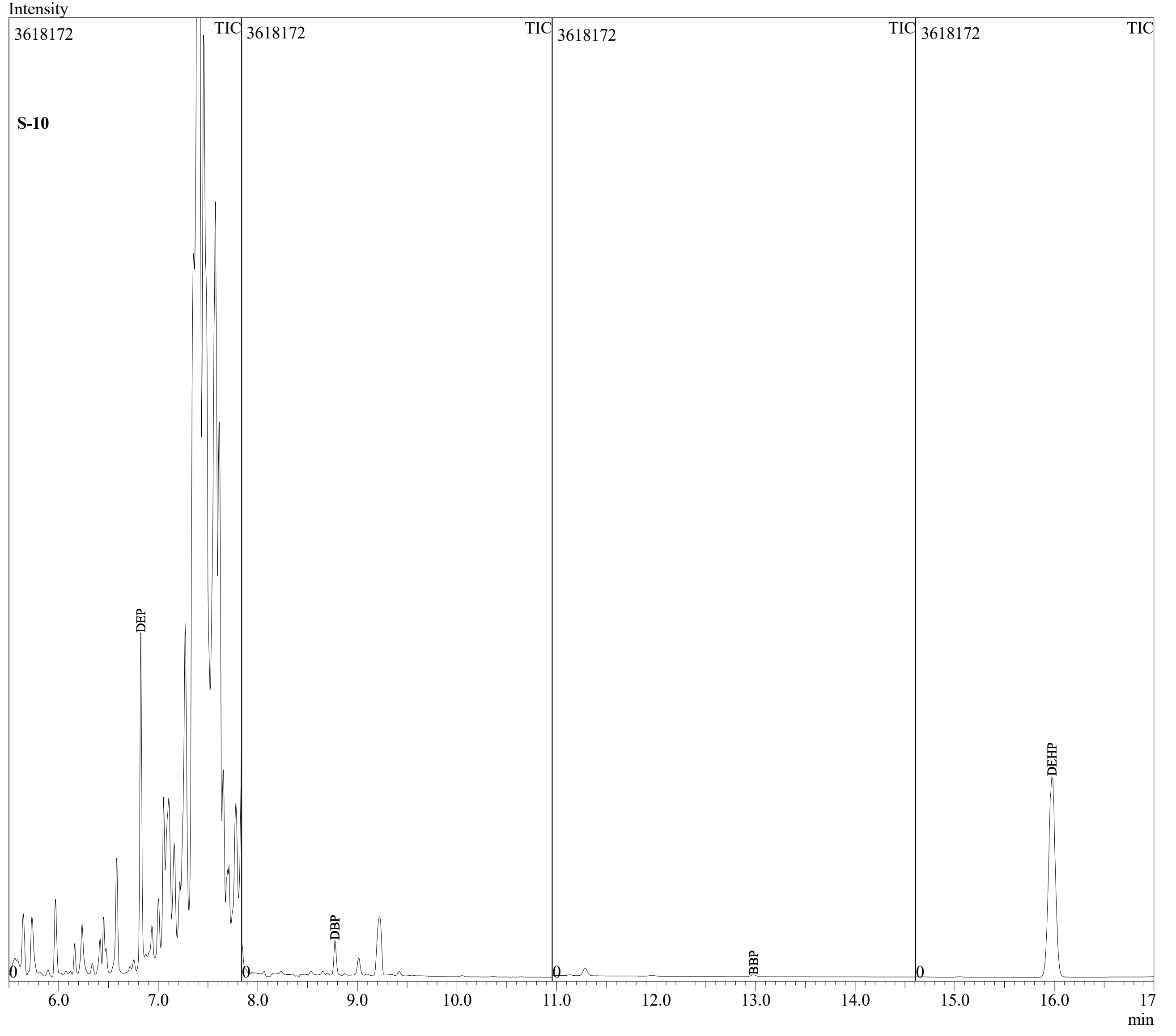
Intensity
3618172
TIC
S-10
DEP
DBP
BBP
DEHP
0
6.0
7.0
8.0
9.0
10.0
11.0
12.0
13.0
14.0
15.0
16.0
17
min

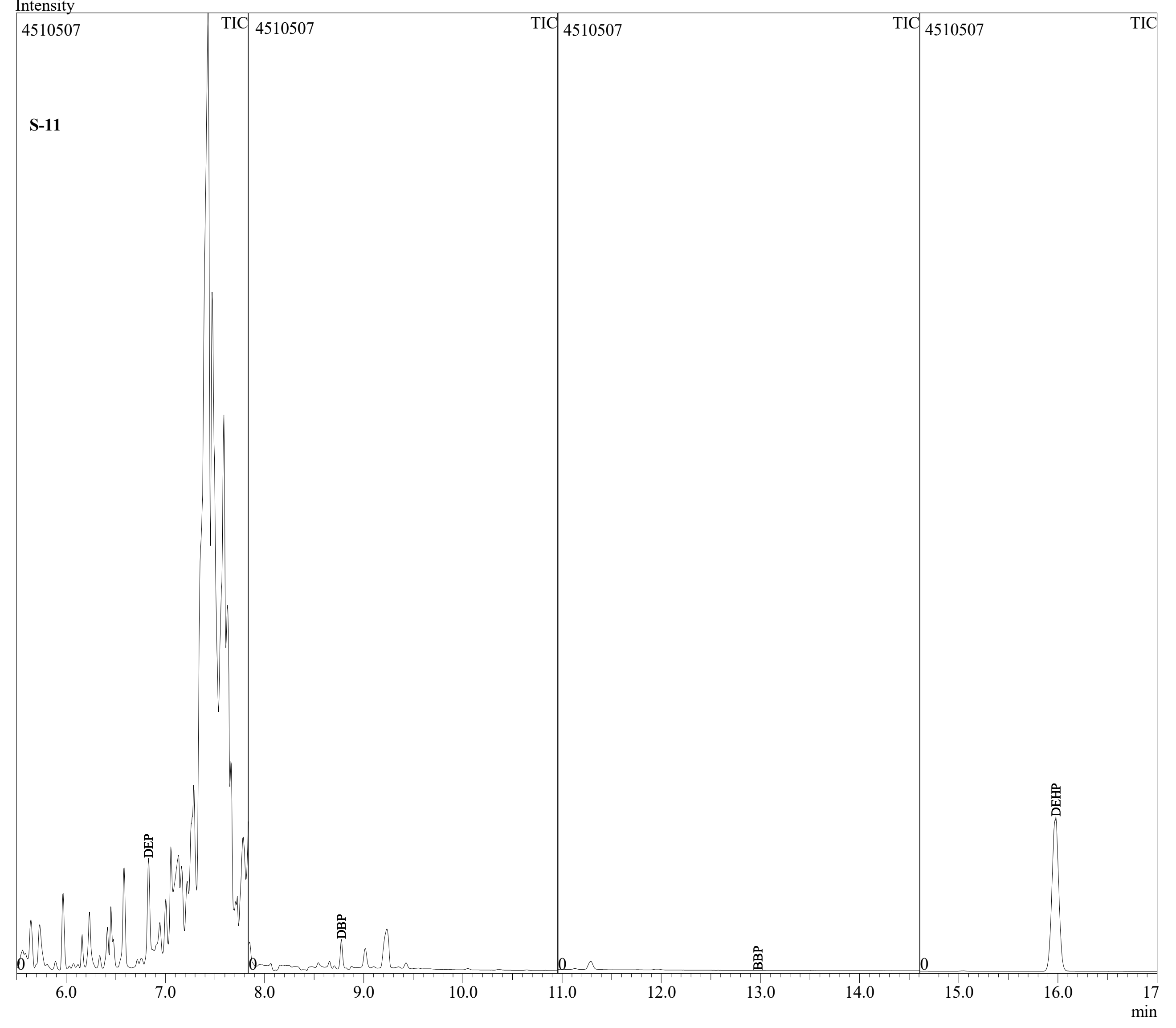
Intensity
4510507
TIC
S-11
DEP
DBP
BBP
DEHP
0
6.0
7.0
8.0
9.0
10.0
11.0
12.0
13.0
14.0
15.0
16.0
17
min

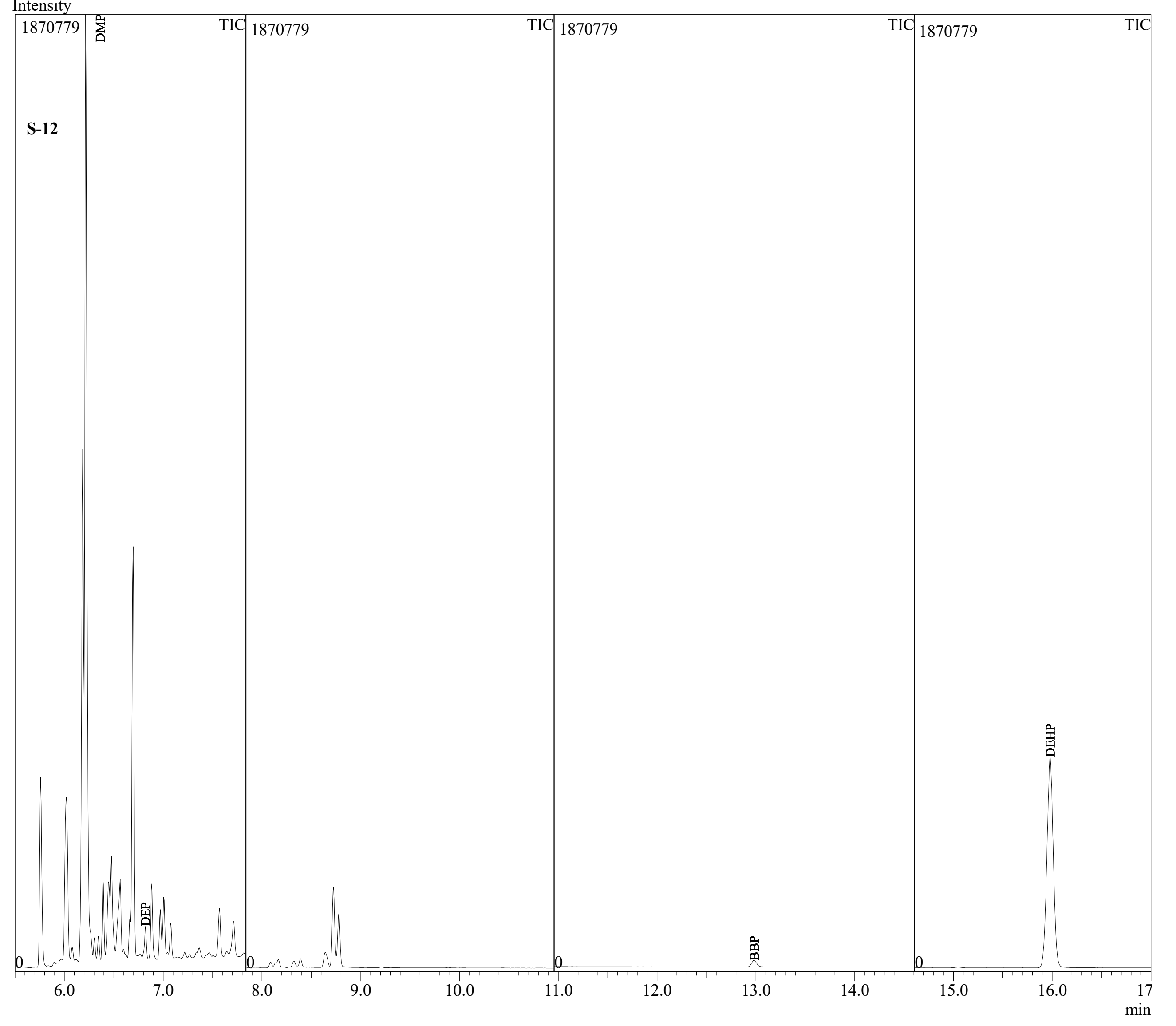
Intensity
1870779
DMP
TIC
S-12
DEP
BBP
DEHP
0
6.0
7.0
8.0
9.0
10.0
11.0
12.0
13.0
14.0
15.0
16.0
17
min

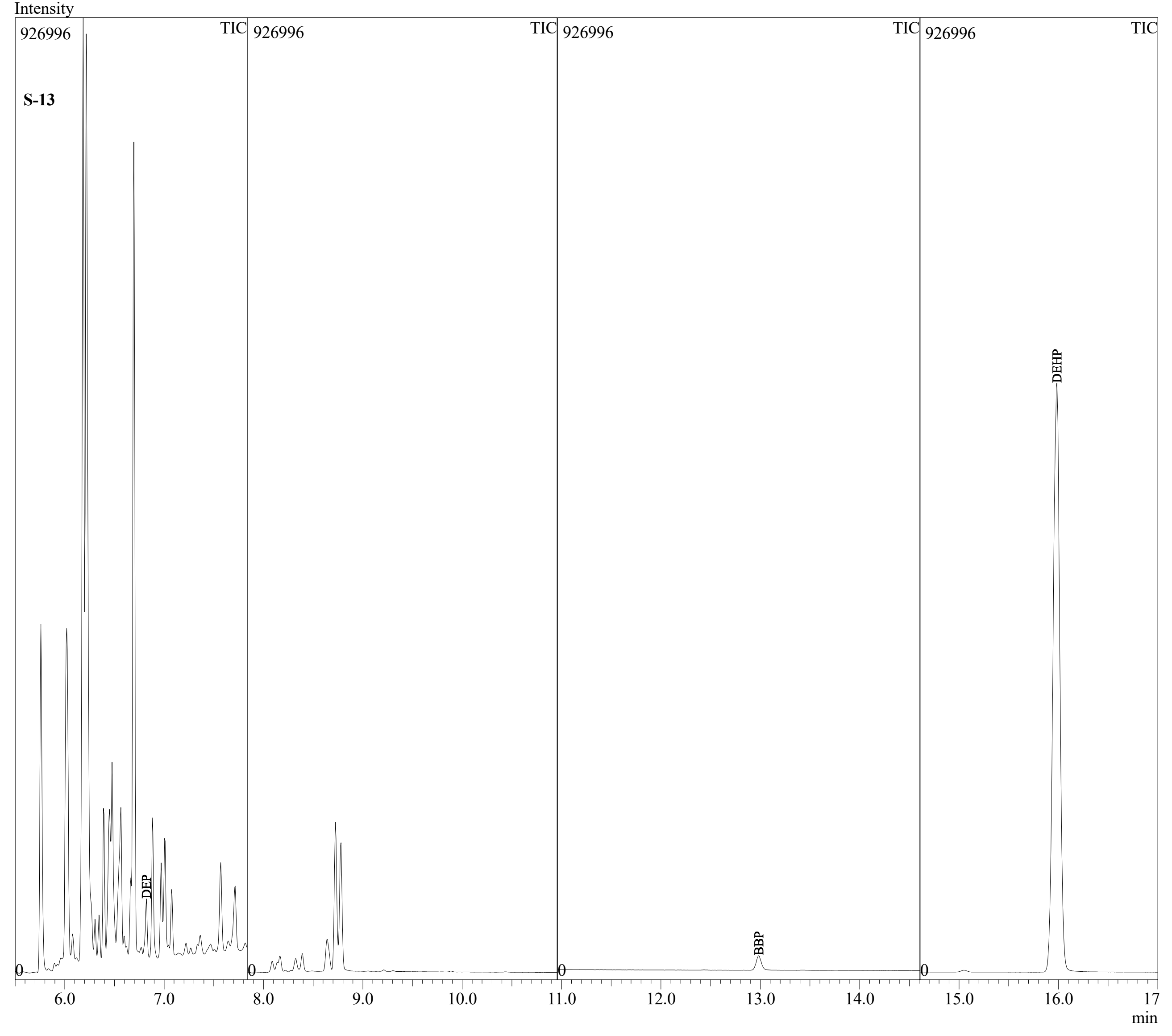

Intensity
926996
TIC
S-13
DEP
BBP
DEHP
0
6.0
7.0
8.0
9.0
10.0
11.0
12.0
13.0
14.0
15.0
16.0
17
min

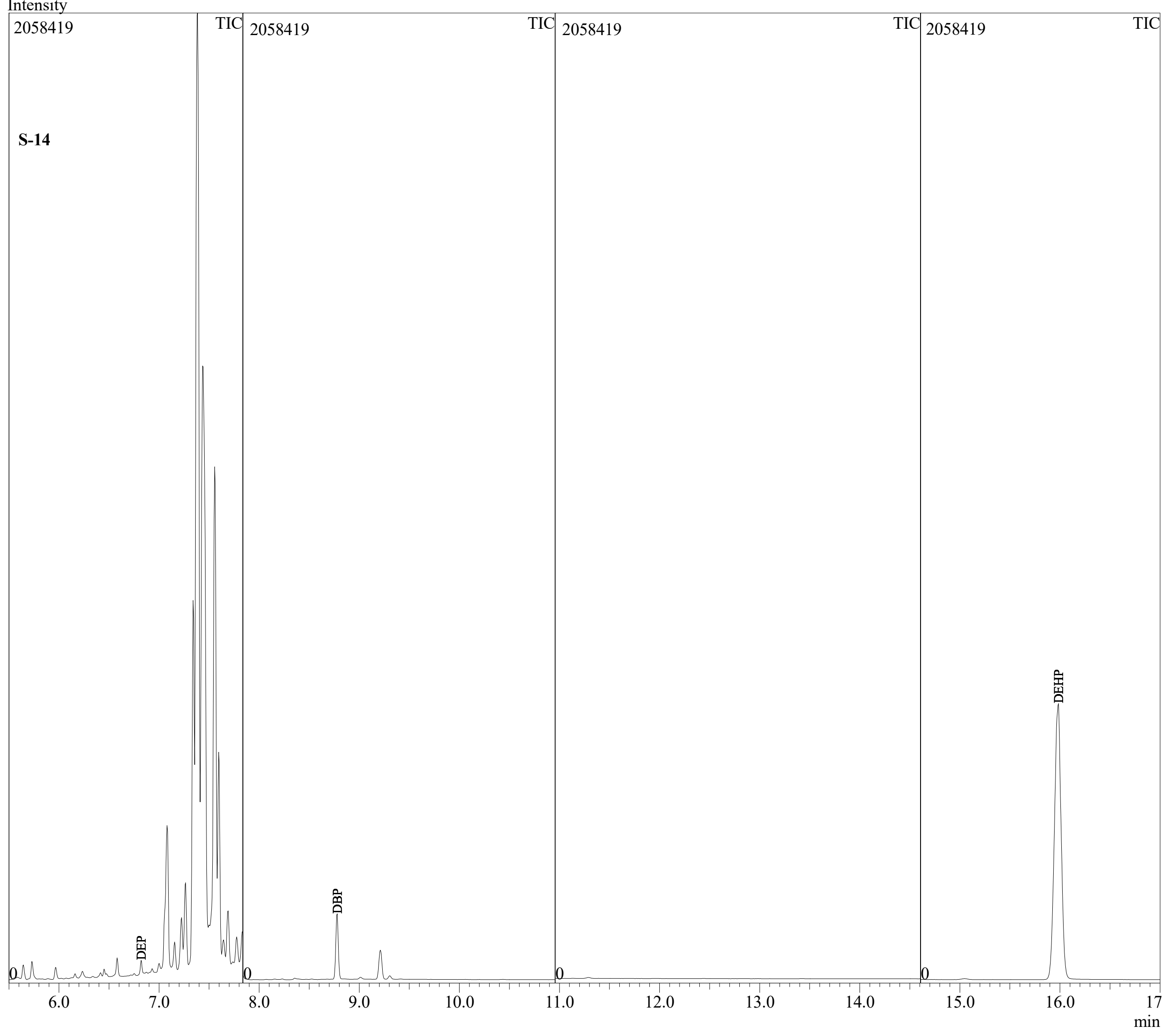
Intensity
2058419
TIC
S-14
DEP
DBP
DEHP
0
6.0
7.0
8.0
9.0
10.0
11.0
12.0
13.0
14.0
15.0
16.0
17
min

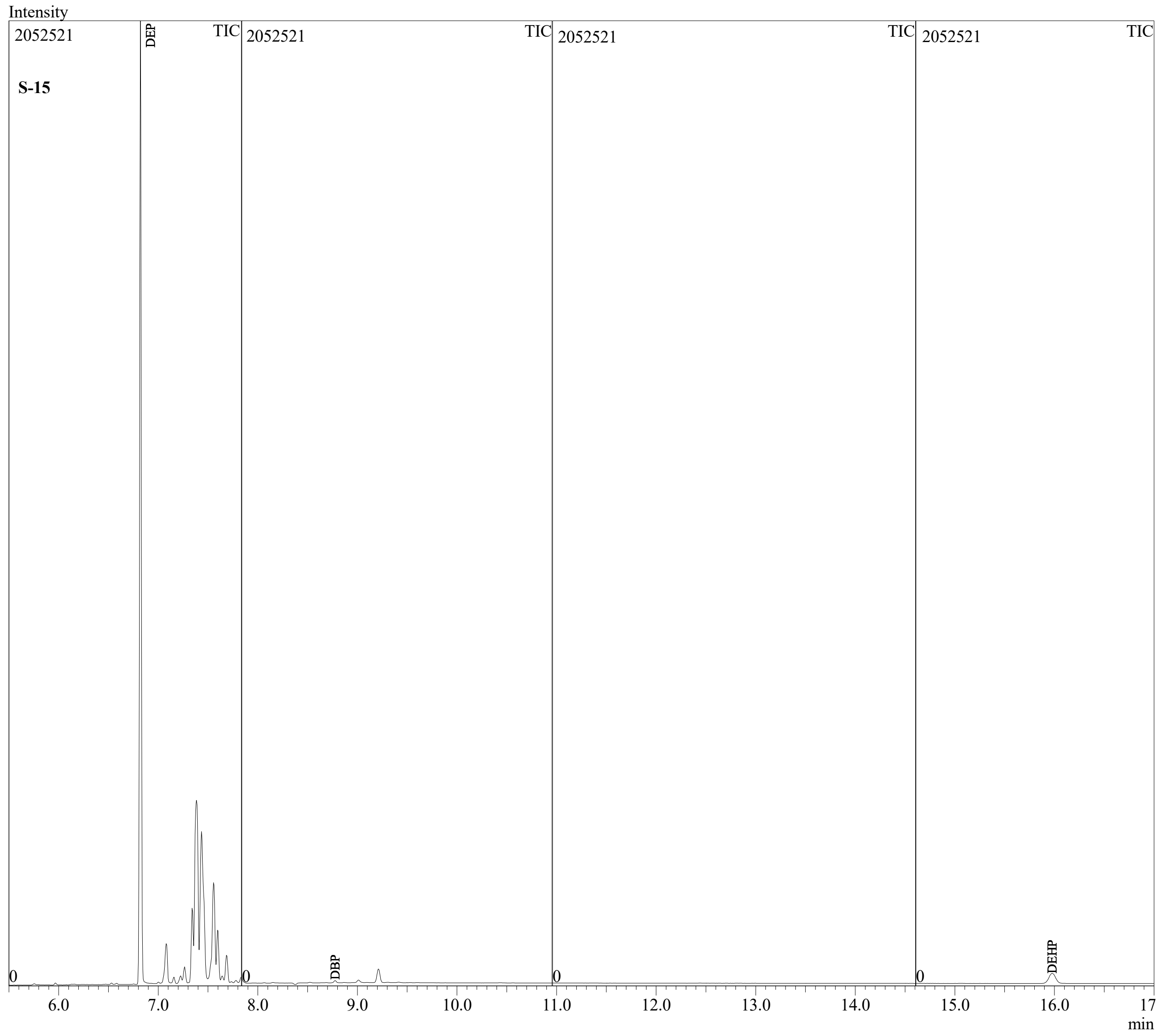

Intensity
2052521
S-15
DEP
TIC
2052521
TIC
2052521
TIC
2052521
TIC
0
DBP
0
0
DEHP
0
6.0
7.0
8.0
9.0
10.0
11.0
12.0
13.0
14.0
15.0
16.0
17
min

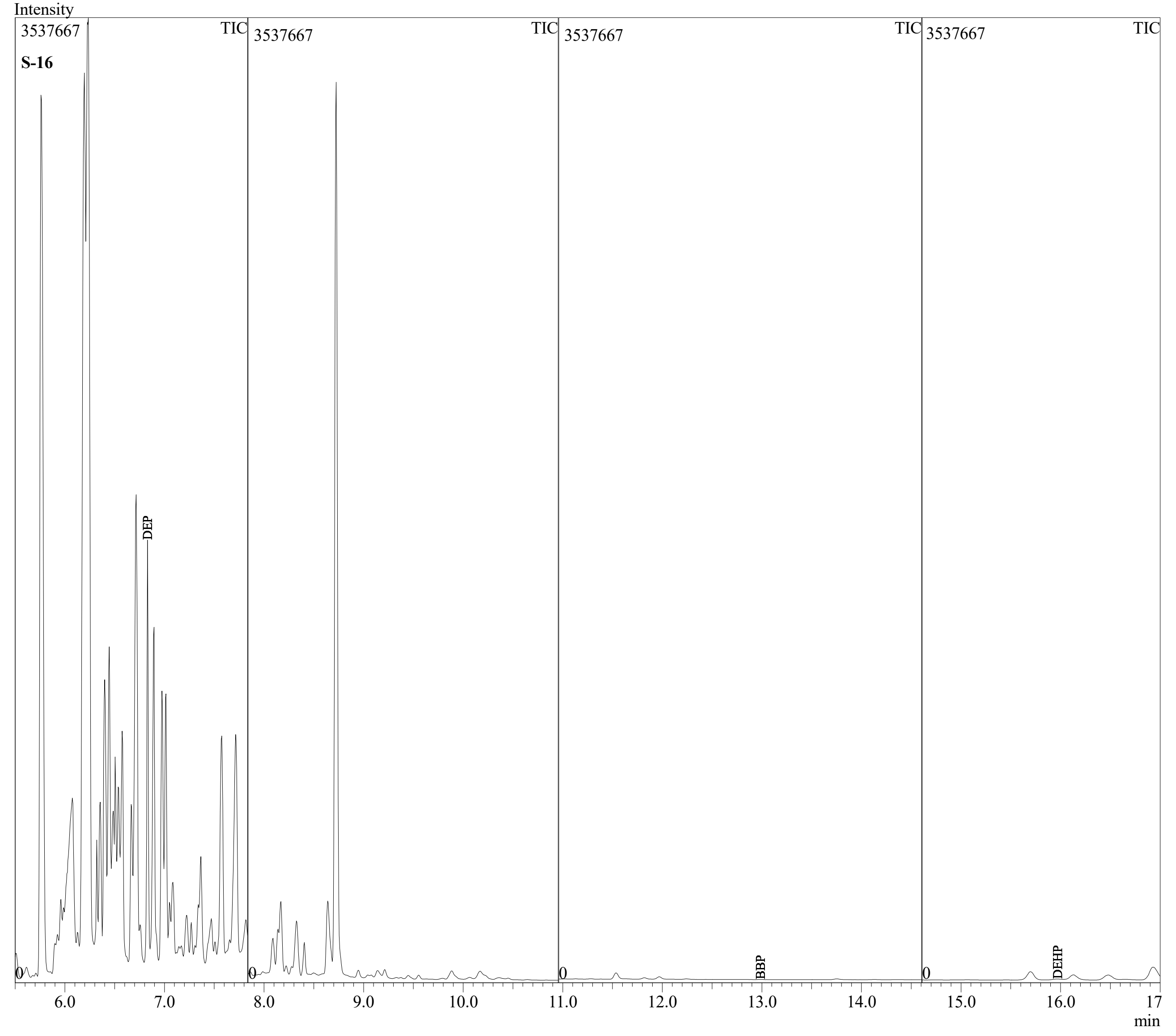
Intensity
3537667
TIC
S-16
DEP
3537667
TIC
3537667
TIC
BBP
3537667
TIC
DEHP
0
0
0
0
6.0
7.0
8.0
9.0
10.0
11.0
12.0
13.0
14.0
15.0
16.0
17
min

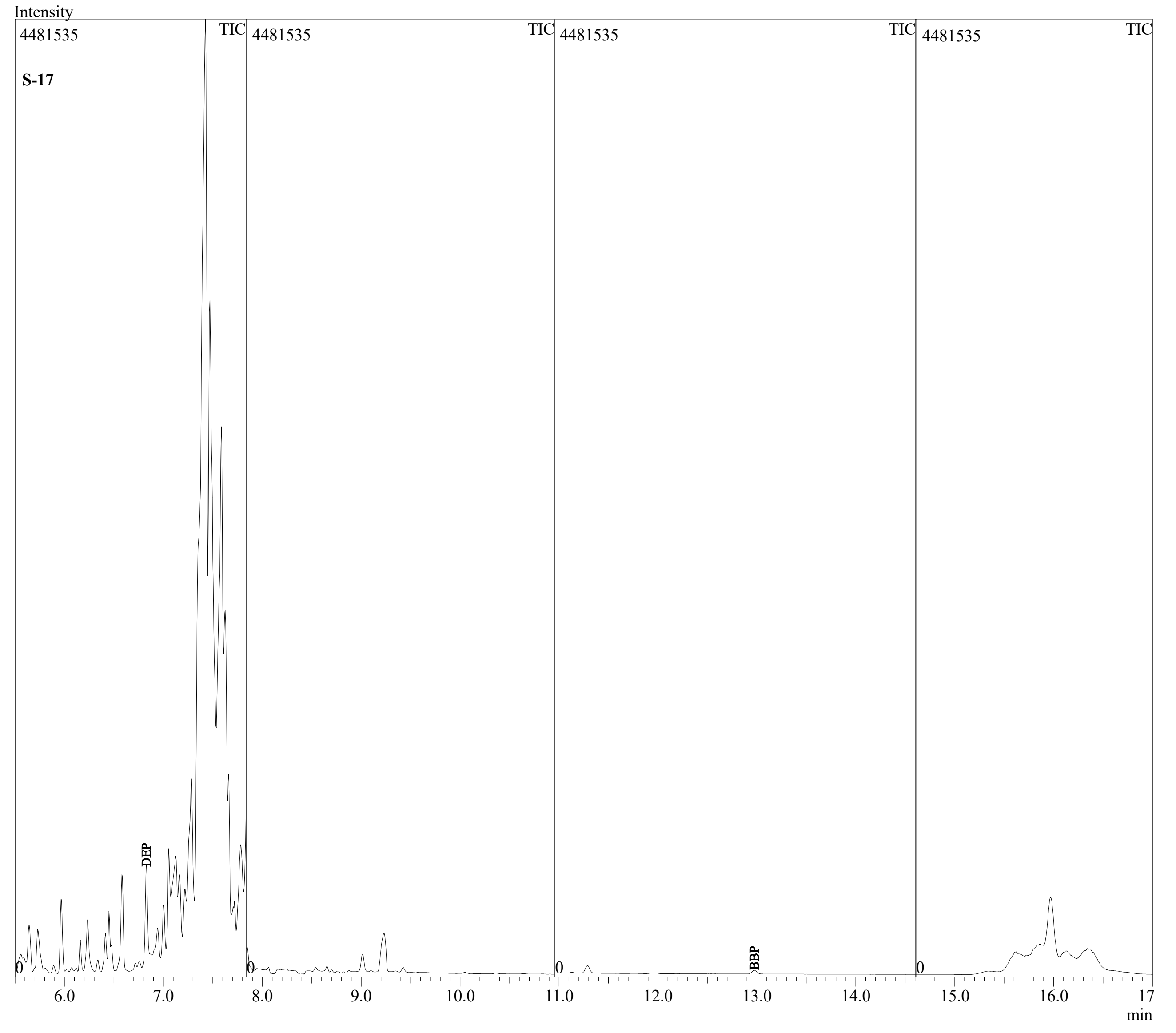

Intensity
4481535
TIC
S-17
DEP
BBP
0
6.0
7.0
8.0
9.0
10.0
11.0
12.0
13.0
14.0
15.0
16.0
17
min

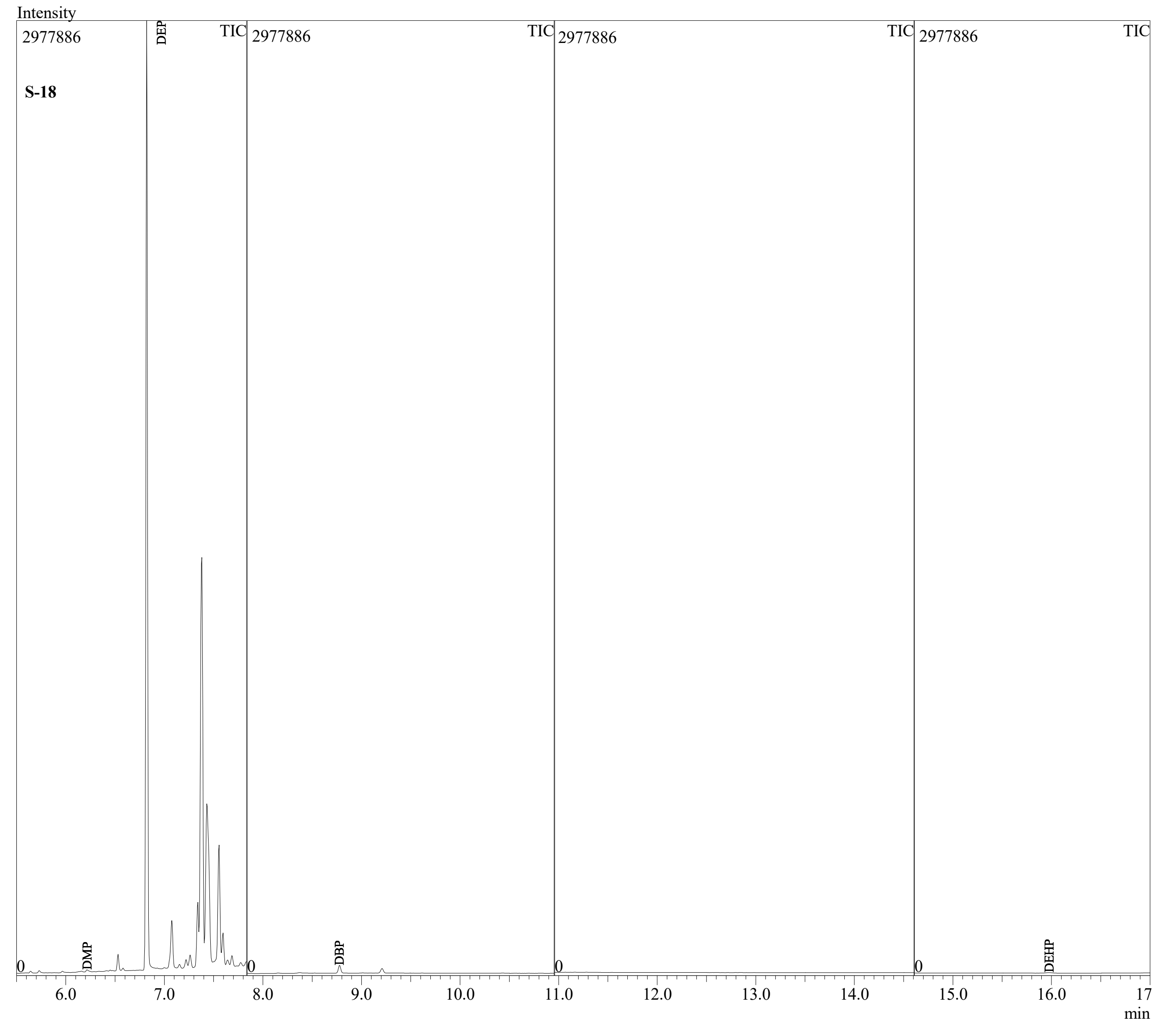

Intensity
2977886
S-18
DMP
DEP
TIC
0
2977886
DBP
TIC
0
2977886
TIC
0
2977886
DEHP
TIC
0
6.0
7.0
8.0
9.0
10.0
11.0
12.0
13.0
14.0
15.0
16.0
17
min

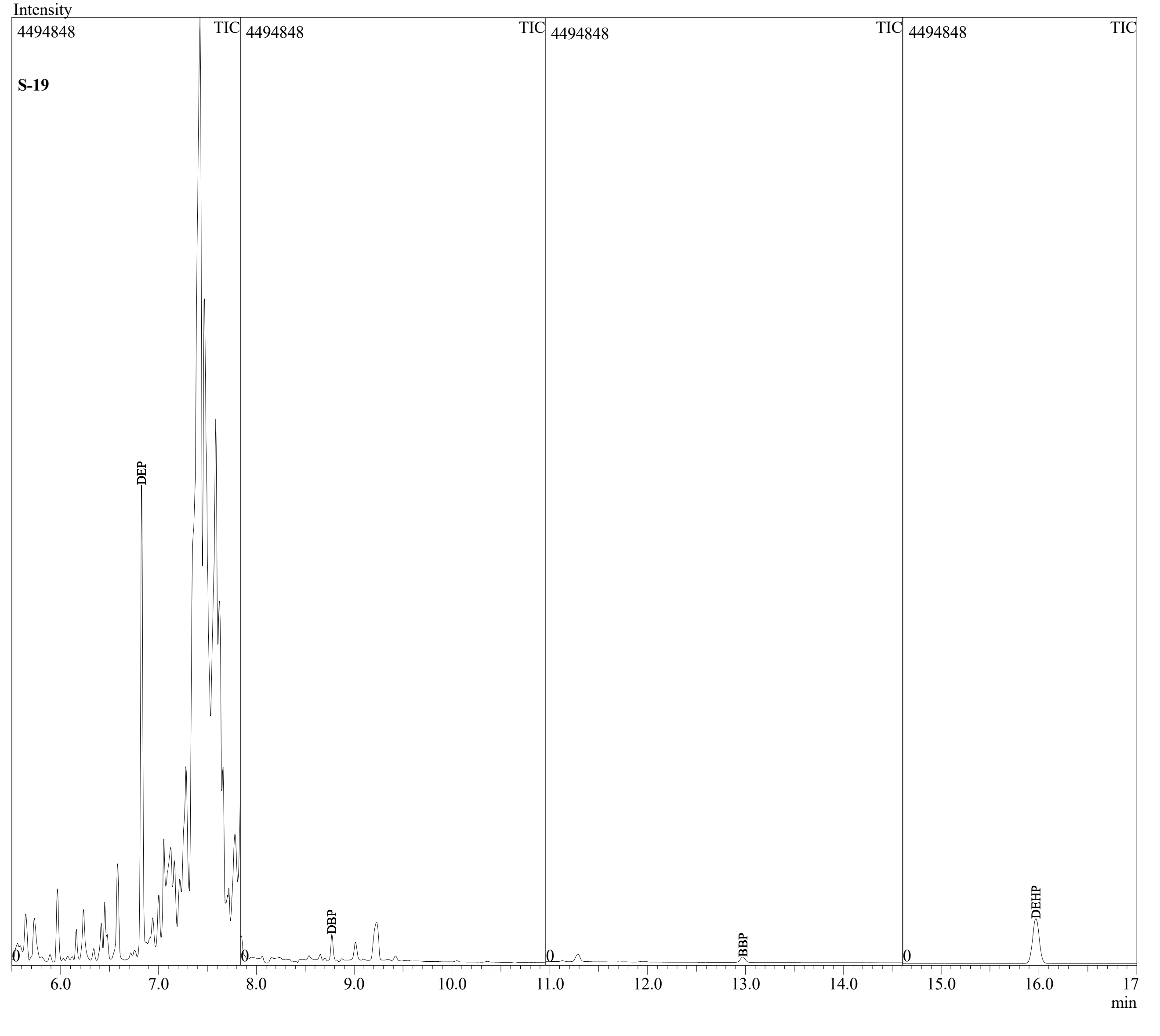
Intensity
4494848
TIC
S-19
DEP
DBP
BBP
DEHP
0
6.0
7.0
8.0
9.0
10.0
11.0
12.0
13.0
14.0
15.0
16.0
17
min

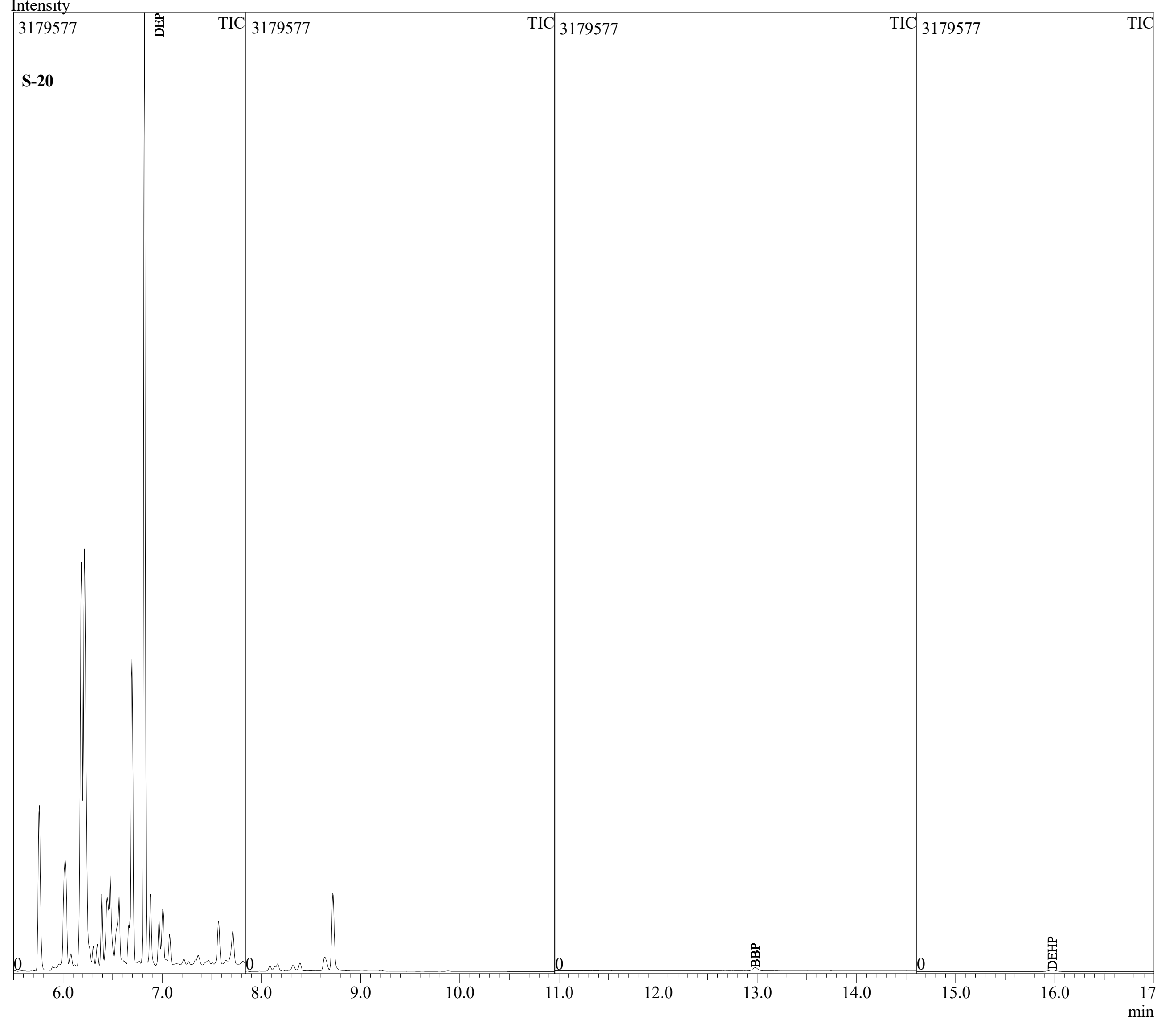
Intensity
3179577
S-20
DEP
TIC
BBP
DEHP
0
6.0
7.0
8.0
9.0
10.0
11.0
12.0
13.0
14.0
15.0
16.0
17
min

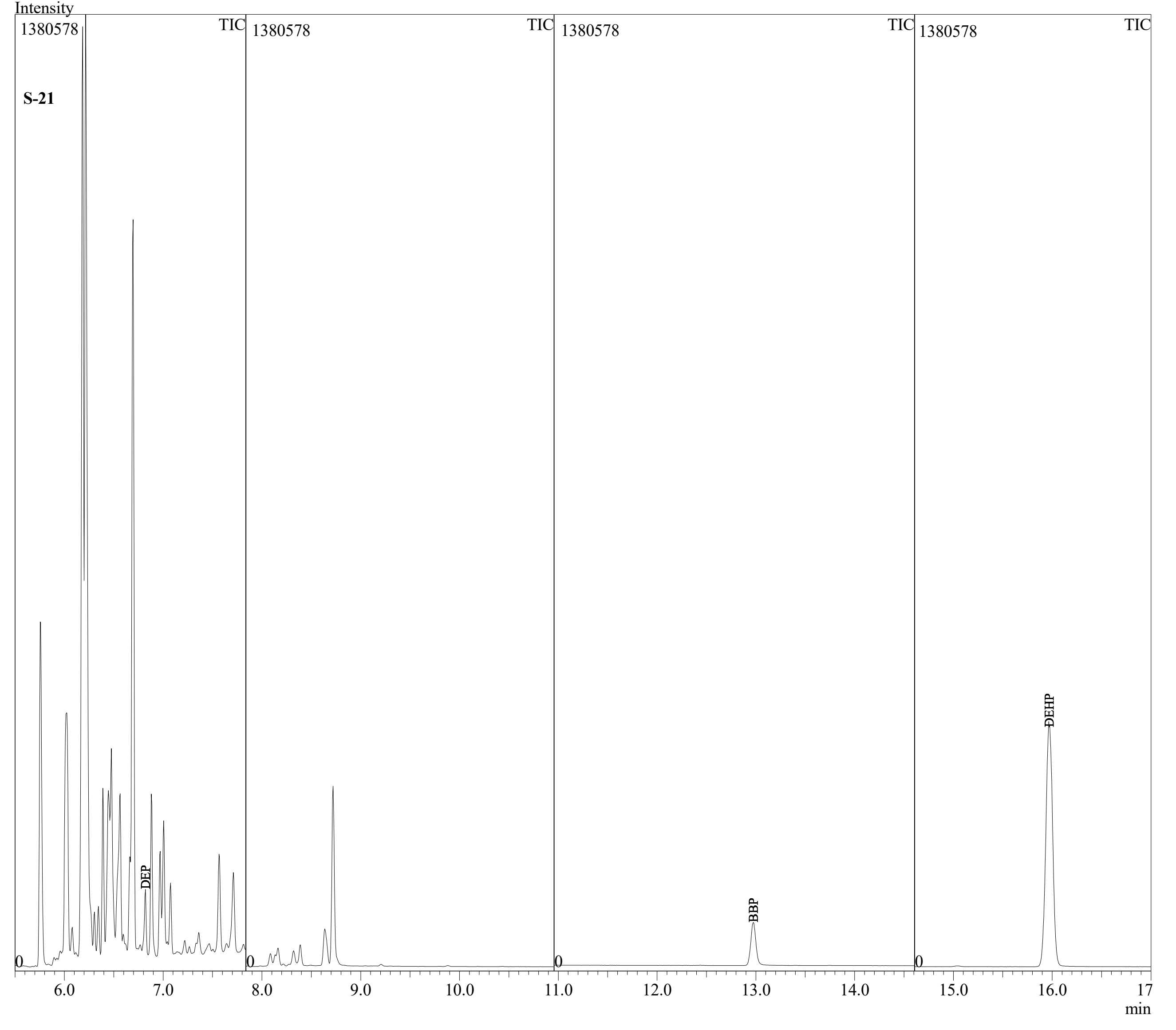

Intensity
1380578
TIC
S-21
DEP
BBP
DEHP
0
6.0
7.0
8.0
9.0
10.0
11.0
12.0
13.0
14.0
15.0
16.0
17
min

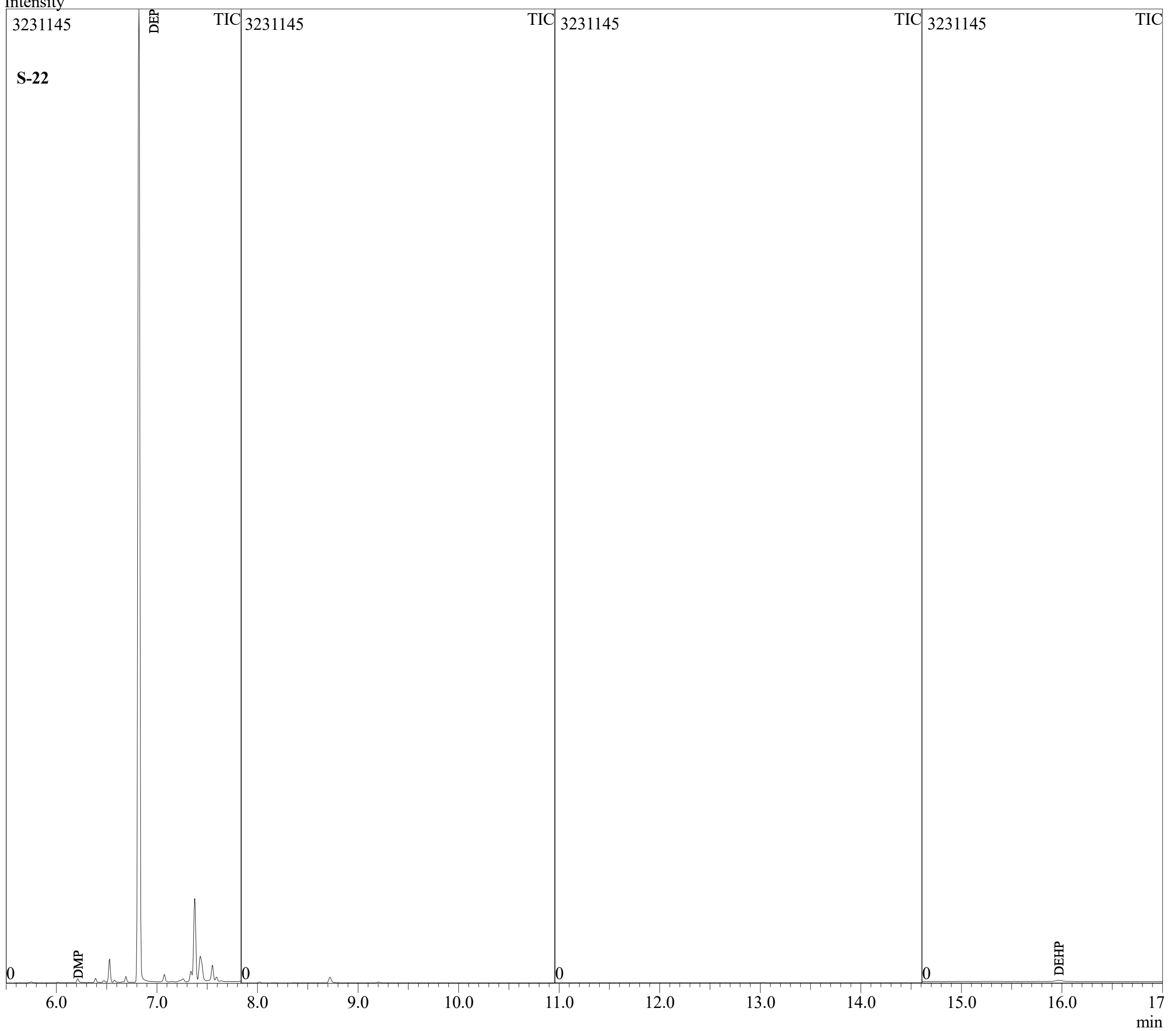
Intensity
3231145
S-22
DMP
DEP
TIC
0
6.0
7.0
8.0
9.0
10.0
11.0
12.0
13.0
14.0
15.0
16.0
17
min
DEHP

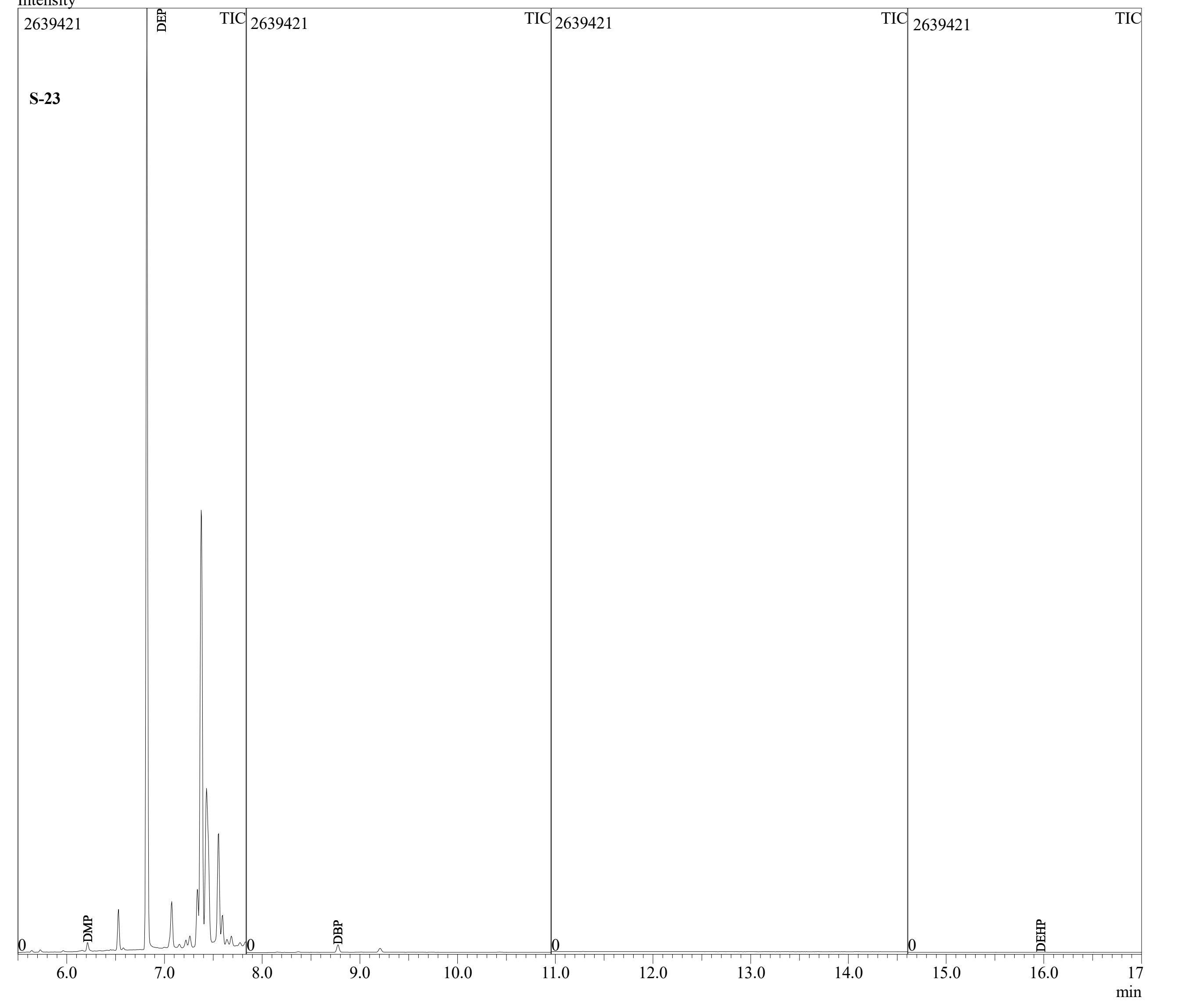
Intensity
2639421
S-23
DMP
DEP
TIC
0
2639421
DBP
TIC
0
2639421
TIC
0
2639421
DEHP
TIC
0
6.0
7.0
8.0
9.0
10.0
11.0
12.0
13.0
14.0
15.0
16.0
17
min

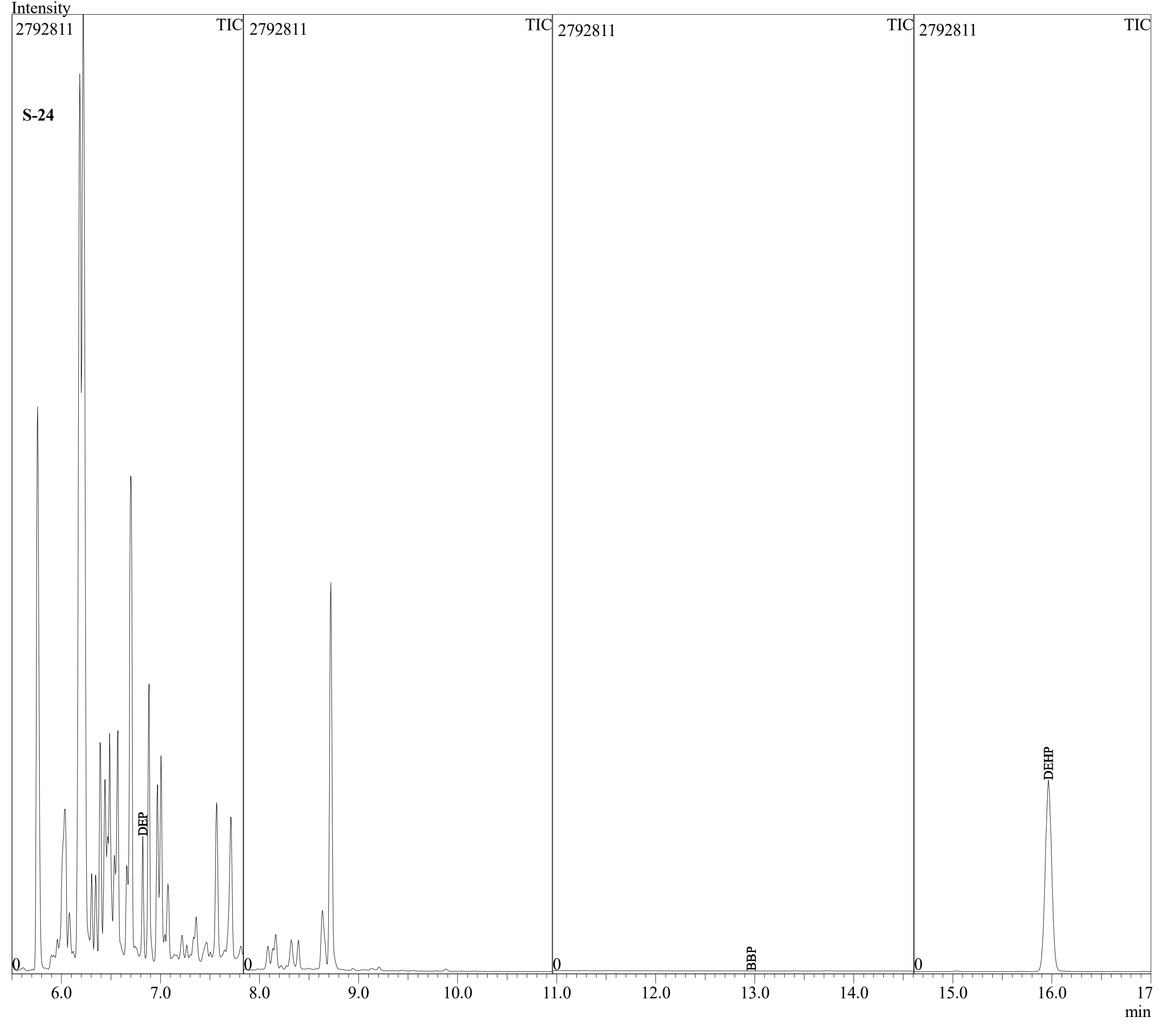
Intensity
2792811
TIC
S-24
DEP
2792811
TIC
2792811
TIC
BBP
2792811
TIC
DEHP
0
6.0
7.0
8.0
9.0
10.0
11.0
12.0
13.0
14.0
15.0
16.0
17
min

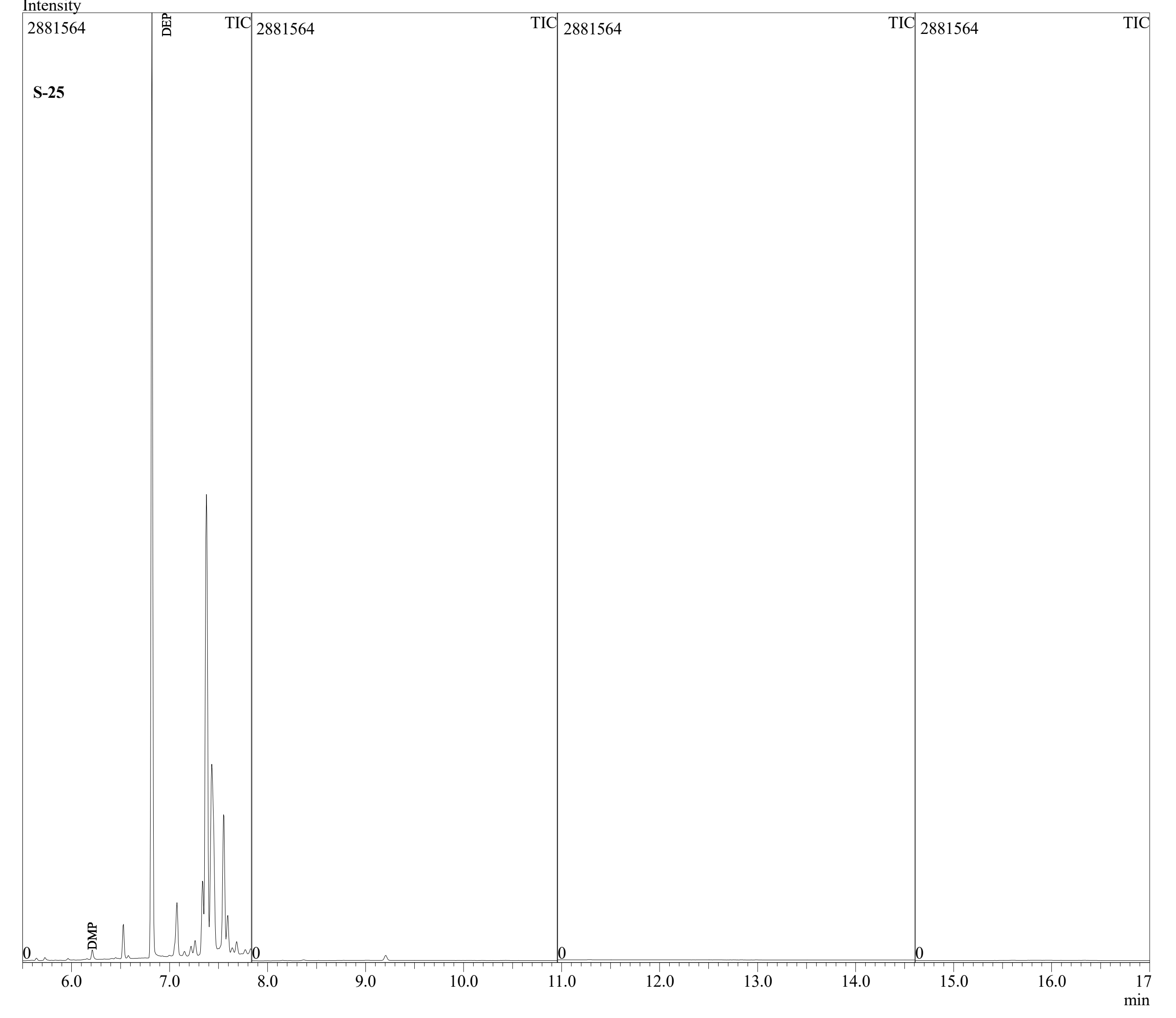

Intensity
2881564
S-25
DMP
DEP
TIC
0
6.0
7.0
8.0
9.0
10.0
11.0
12.0
13.0
14.0
15.0
16.0
17
min

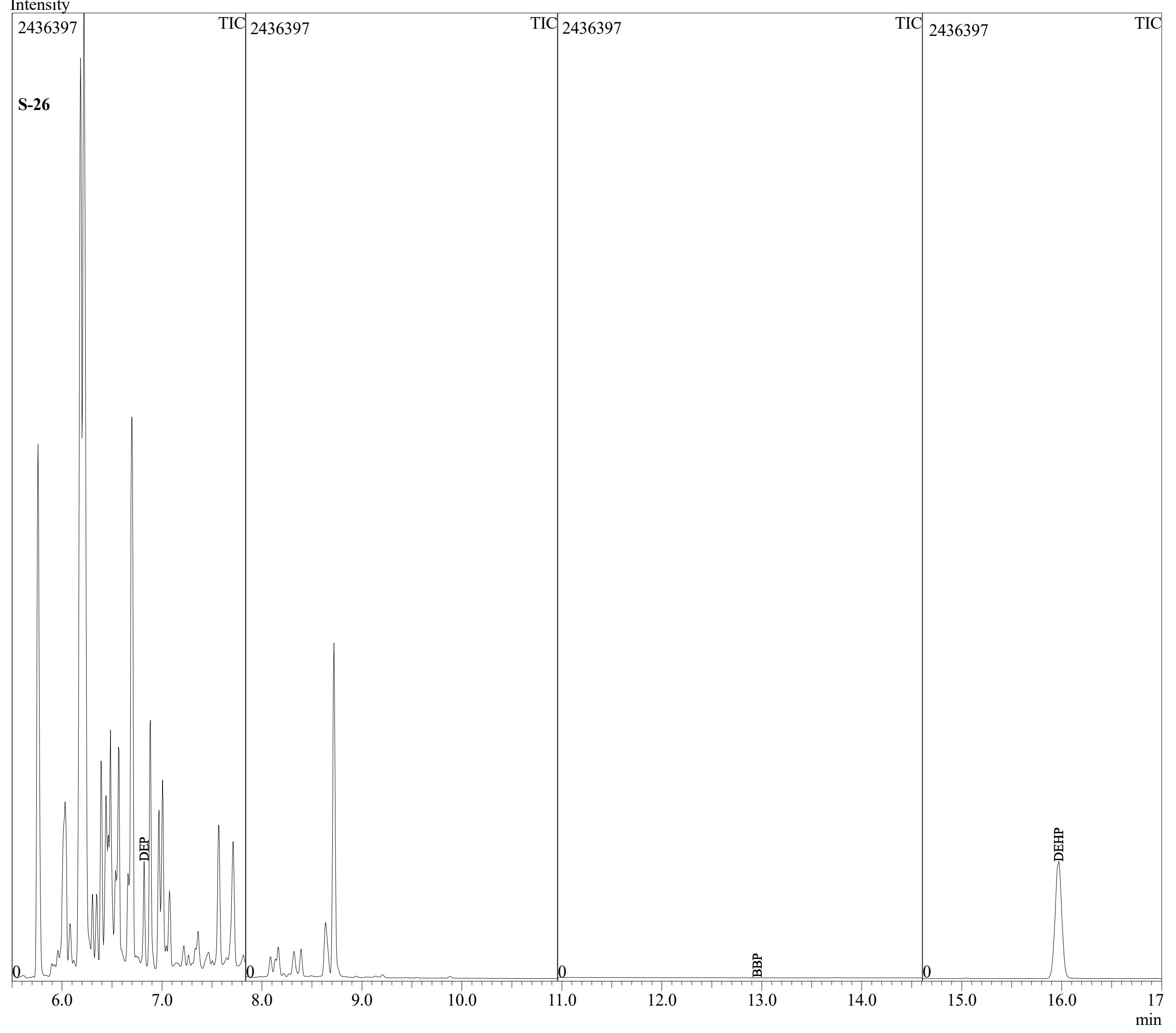

Intensity
2436397
TIC
S-26
DEP
BBP
DEHP
0
6.0
7.0
8.0
9.0
10.0
11.0
12.0
13.0
14.0
15.0
16.0
17
min

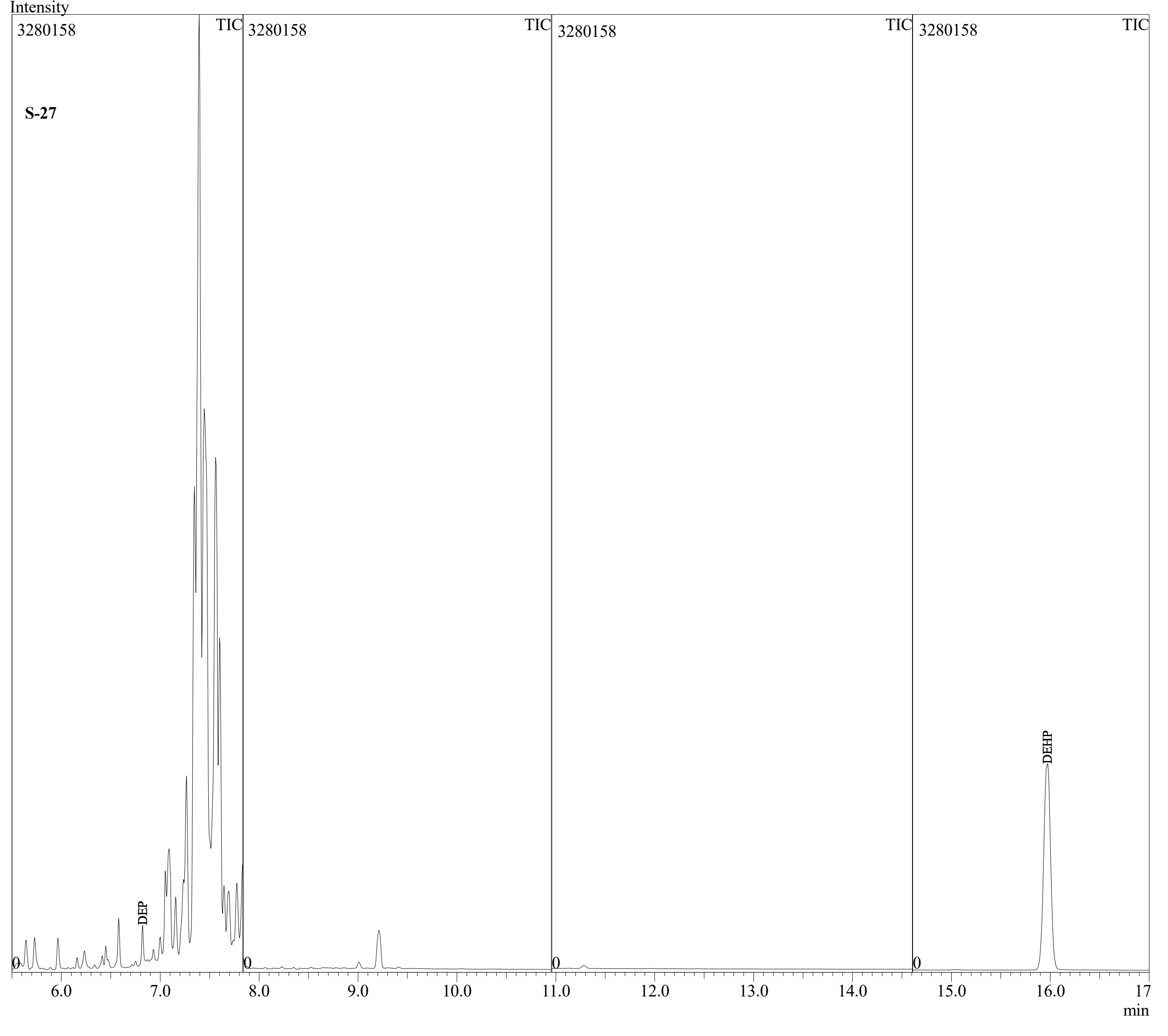
Intensity
3280158
TIC
S-27
DEP
DEHP
0
6.0
7.0
8.0
9.0
10.0
11.0
12.0
13.0
14.0
15.0
16.0
17
min

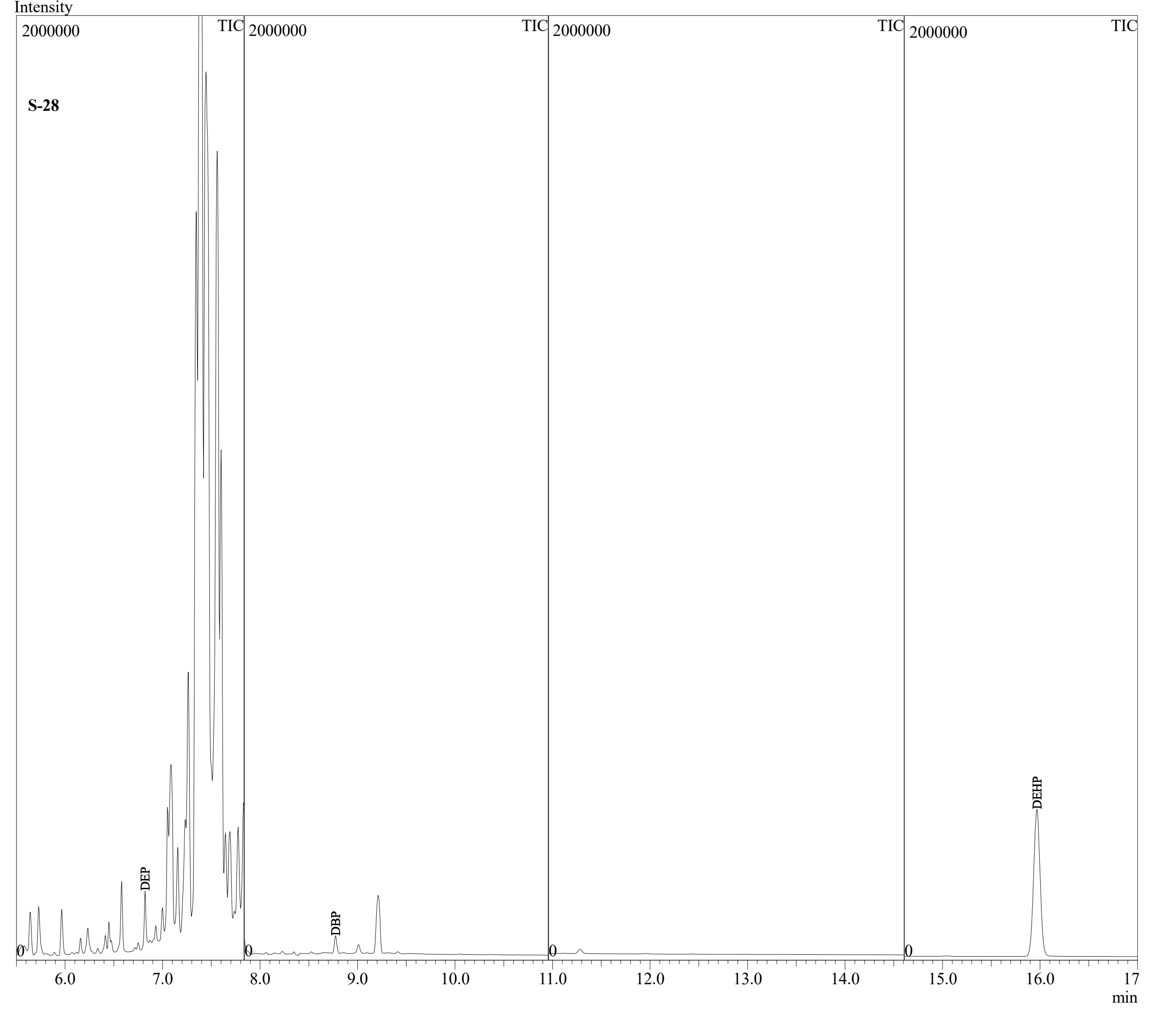
Intensity
2000000
TIC
S-28
DEP
DBP
DEHP
0
6.0
7.0
8.0
9.0
10.0
11.0
12.0
13.0
14.0
15.0
16.0
17
min

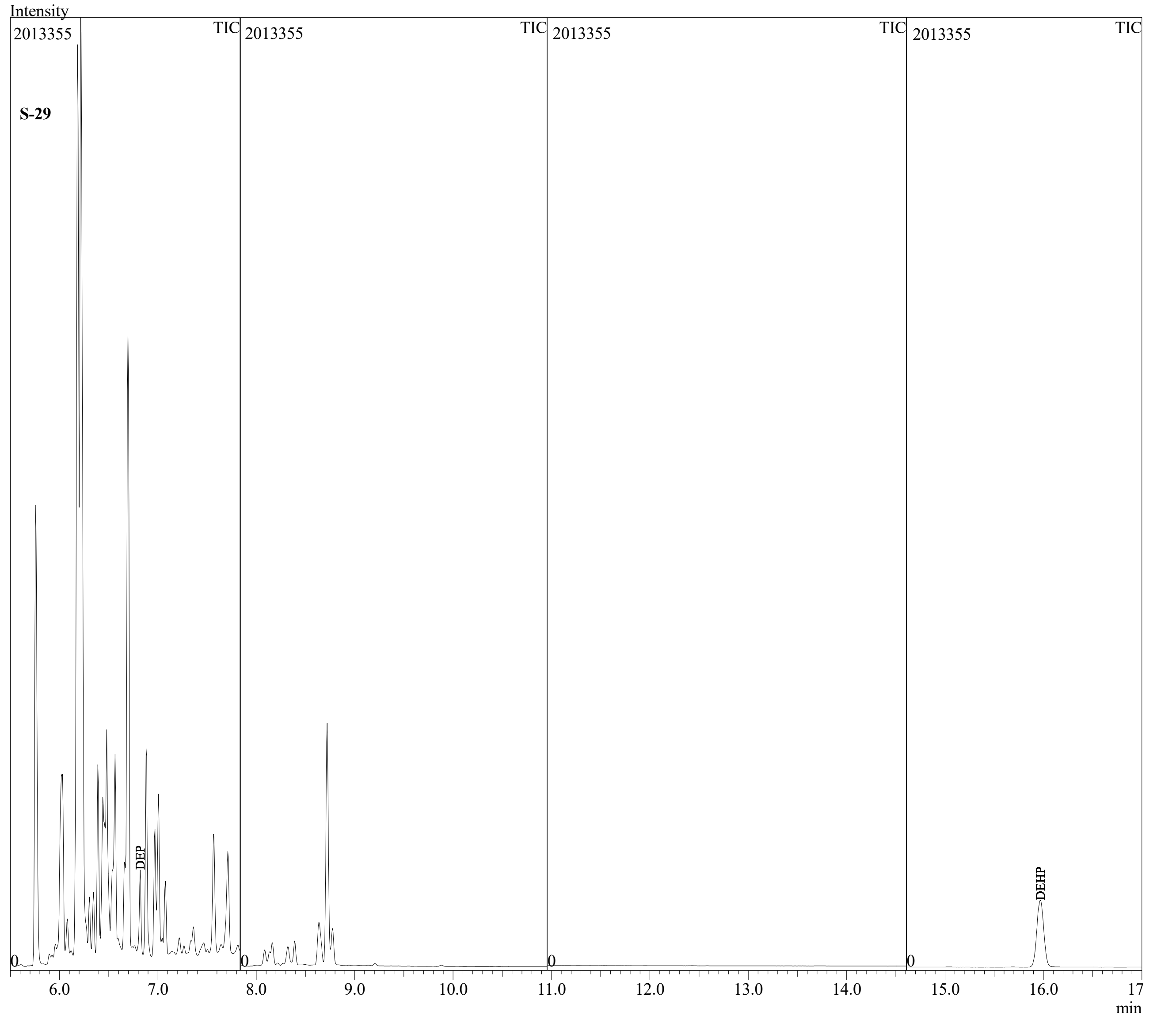
Intensity
2013355
TIC
S-29
DEP
DEHP
0
6.0
7.0
8.0
9.0
10.0
11.0
12.0
13.0
14.0
15.0
16.0
17
min

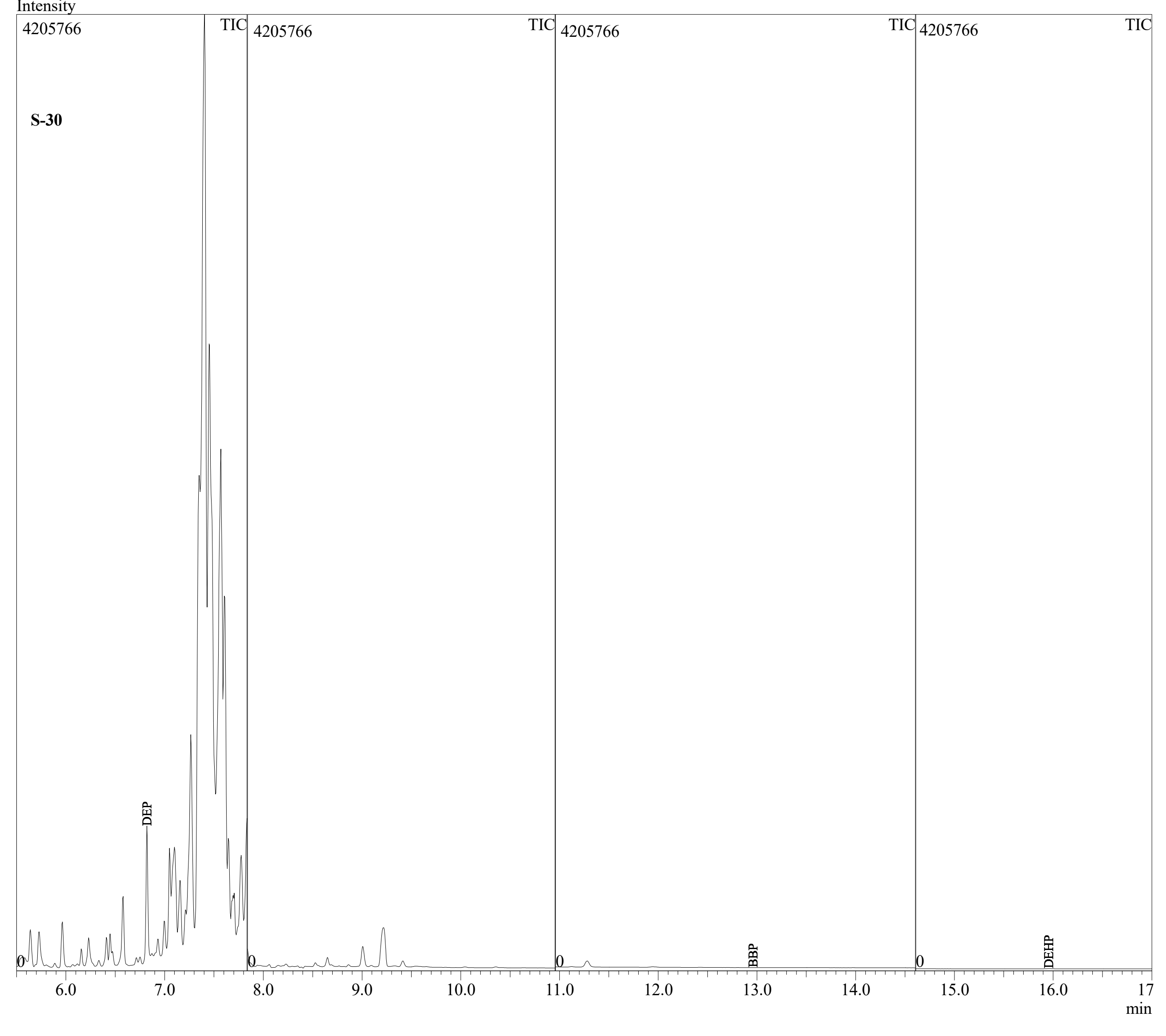

Intensity
4205766
TIC
S-30
DEP
4205766
TIC
4205766
TIC
BBP
4205766
TIC
DEHP
0
6.0
7.0
8.0
9.0
10.0
11.0
12.0
13.0
14.0
15.0
16.0
17
min

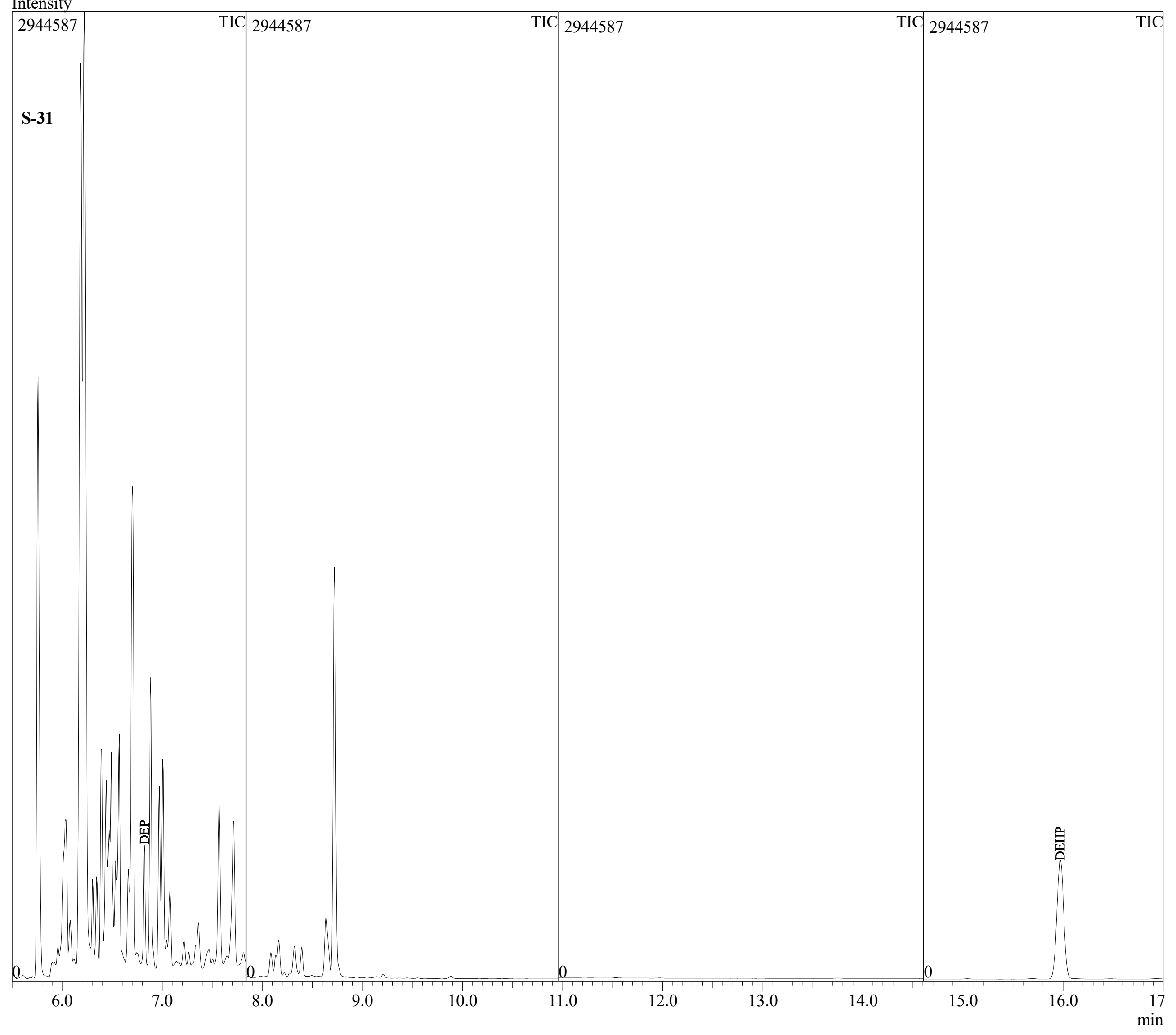
Intensity
2944587
TIC
S-31
DEP
DEHP
0
6.0
7.0
8.0
9.0
10.0
11.0
12.0
13.0
14.0
15.0
16.0
17
min

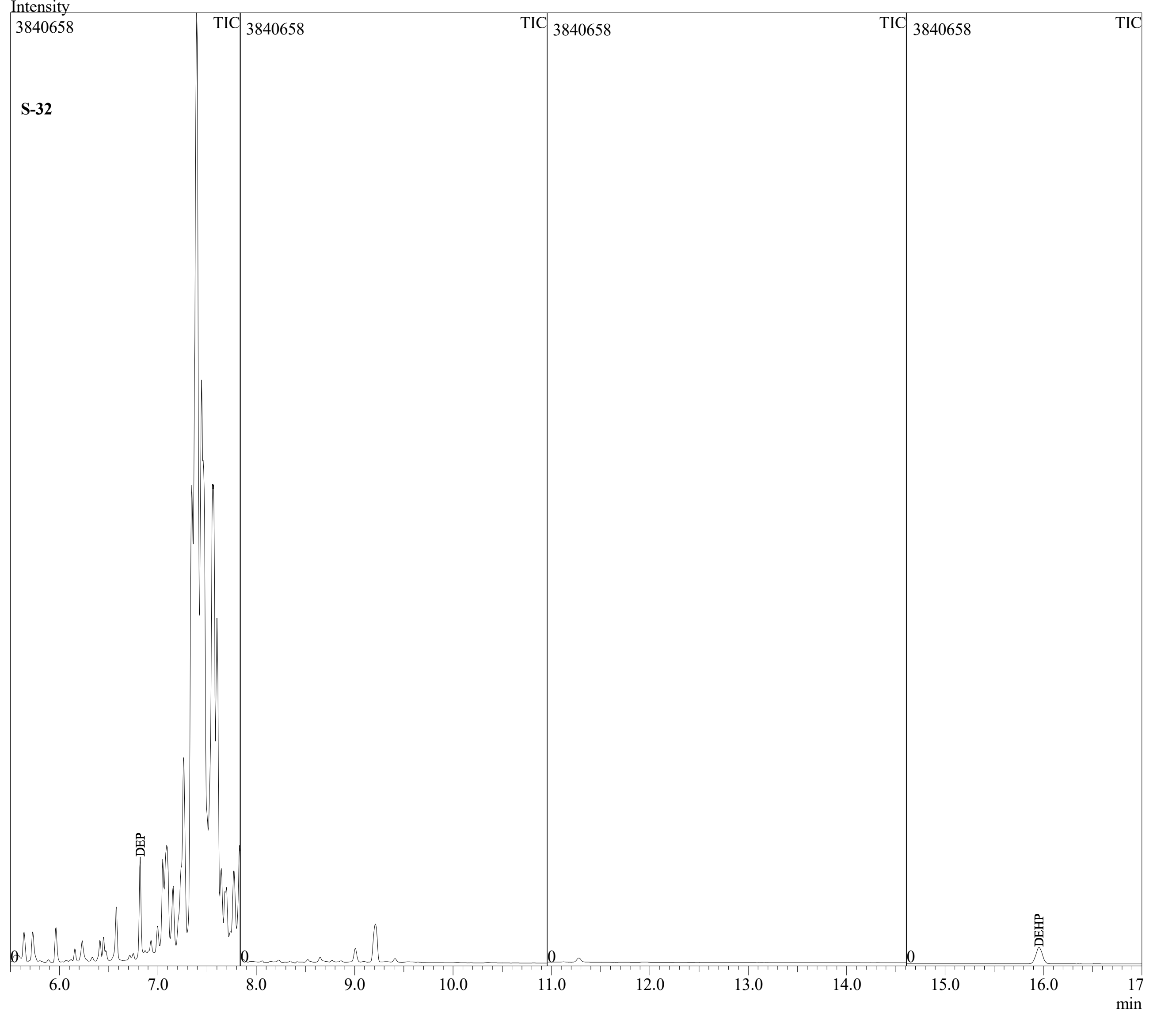

Intensity
3840658
TIC
S-32
DEP
DEHP
0
6.0
7.0
8.0
9.0
10.0
11.0
12.0
13.0
14.0
15.0
16.0
17
min

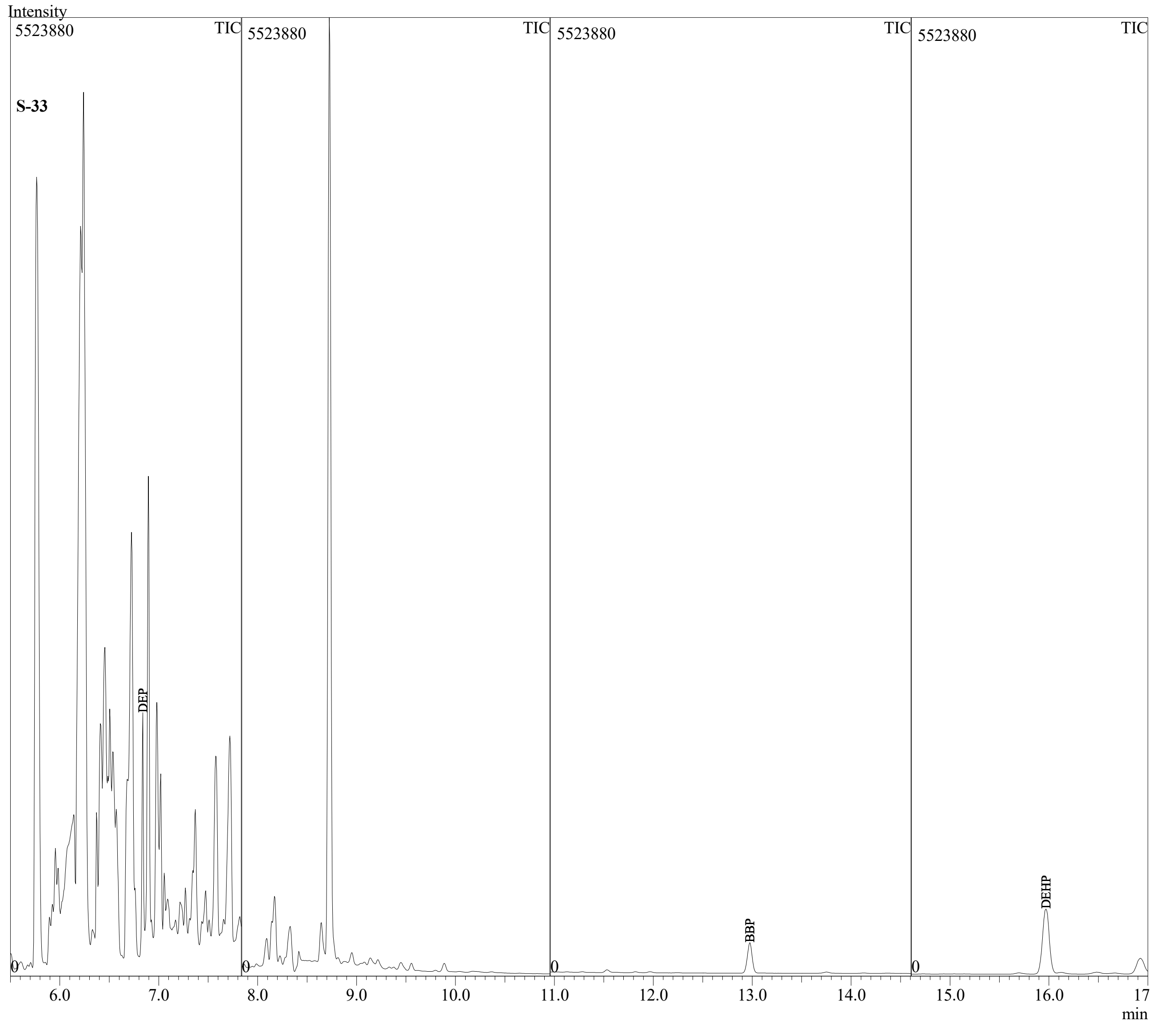
Intensity
5523880
TIC
S-33
DEP
BBP
DEHP
0
6.0
7.0
8.0
9.0
10.0
11.0
12.0
13.0
14.0
15.0
16.0
17
min

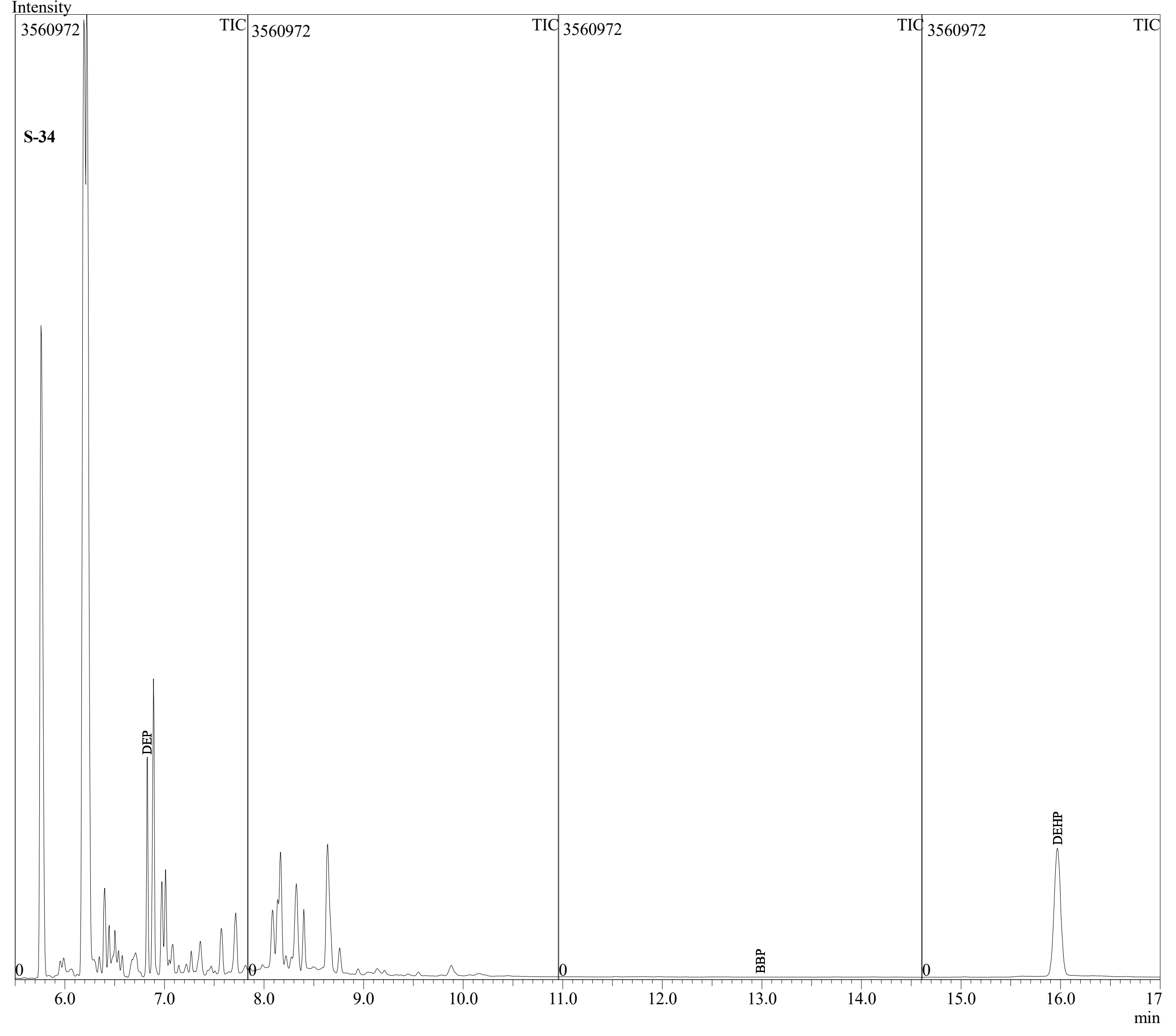

Intensity
3560972
TIC
S-34
DEP
BBP
DEHP
0
6.0
7.0
8.0
9.0
10.0
11.0
12.0
13.0
14.0
15.0
16.0
17
min

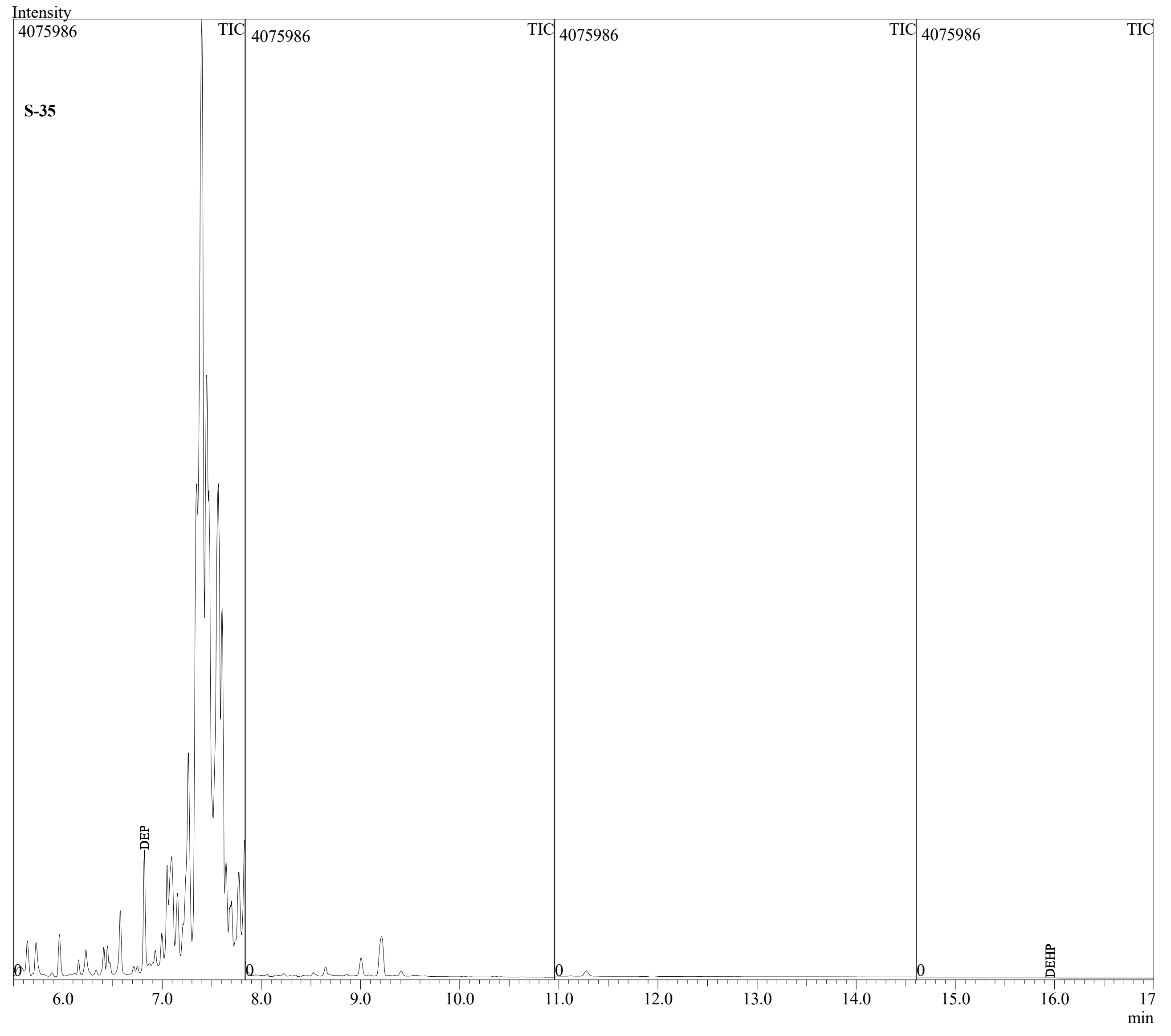

Intensity
4075986
TIC
S-35
DEP
DEHP
0
6.0
7.0
8.0
9.0
10.0
11.0
12.0
13.0
14.0
15.0
16.0
17
min

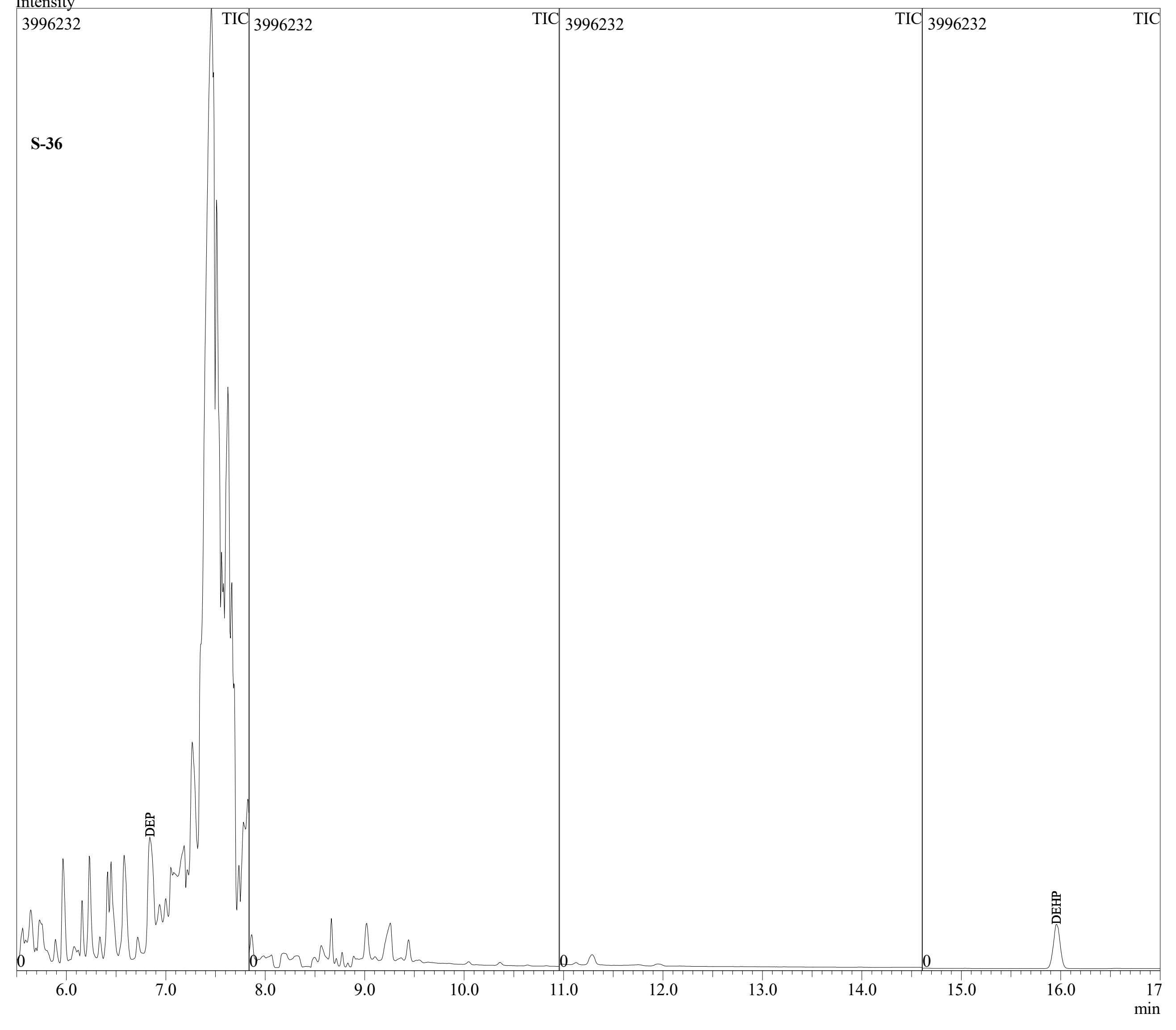
Intensity
3996232
TIC
S-36
DEP
DEHP
0
6.0
7.0
8.0
9.0
10.0
11.0
12.0
13.0
14.0
15.0
16.0
17
min

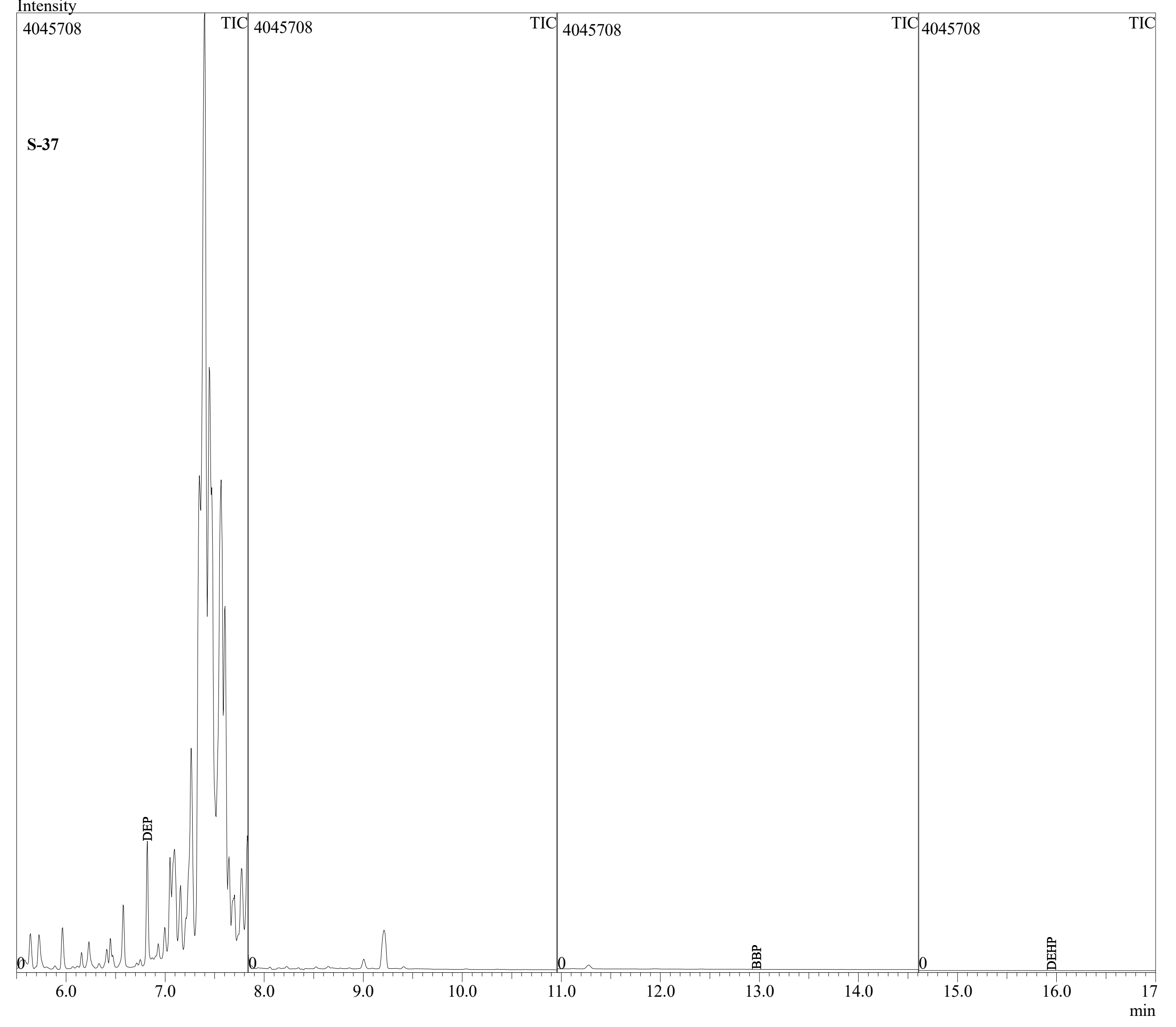
Intensity
4045708
TIC
S-37
DEP
BBP
DEHP
0
6.0
7.0
8.0
9.0
10.0
11.0
12.0
13.0
14.0
15.0
16.0
17
min

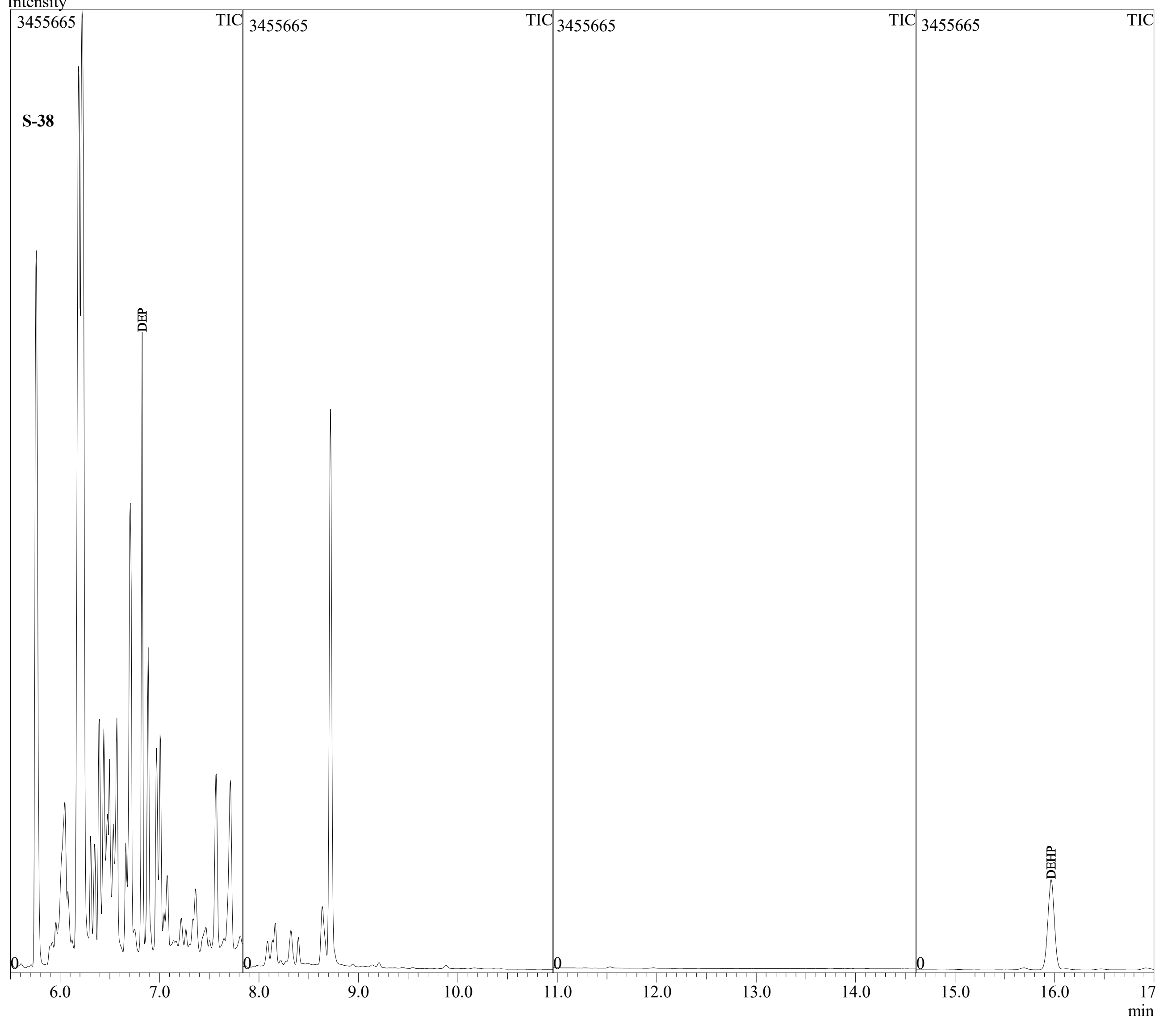
Intensity
3455665
TIC
S-38
DEP
DEHP
0
6.0
7.0
8.0
9.0
10.0
11.0
12.0
13.0
14.0
15.0
16.0
17
min

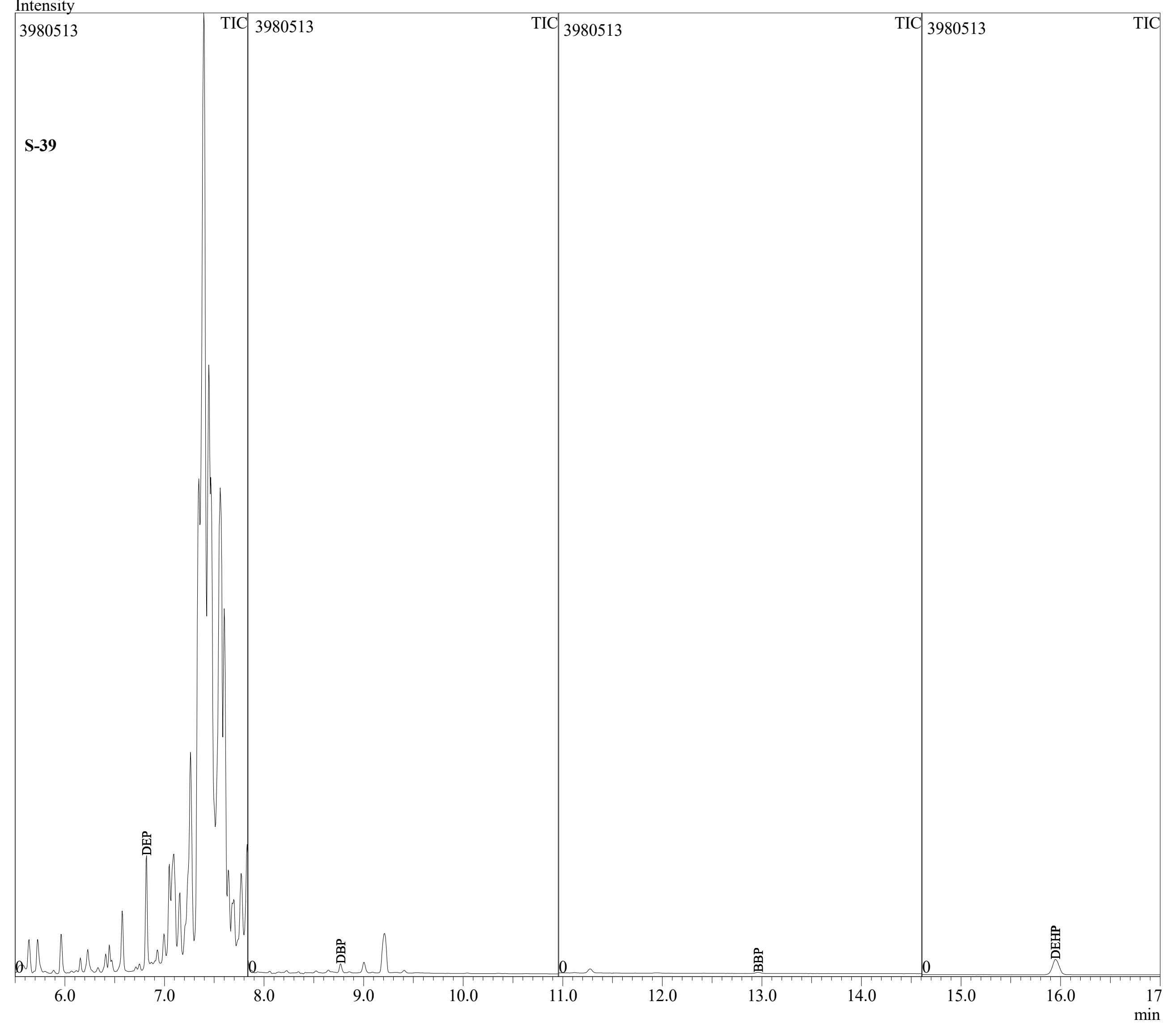

Intensity
3980513
TIC
S-39
DEP
DBP
BBP
DEHP
0
6.0
7.0
8.0
9.0
10.0
11.0
12.0
13.0
14.0
15.0
16.0
17
min

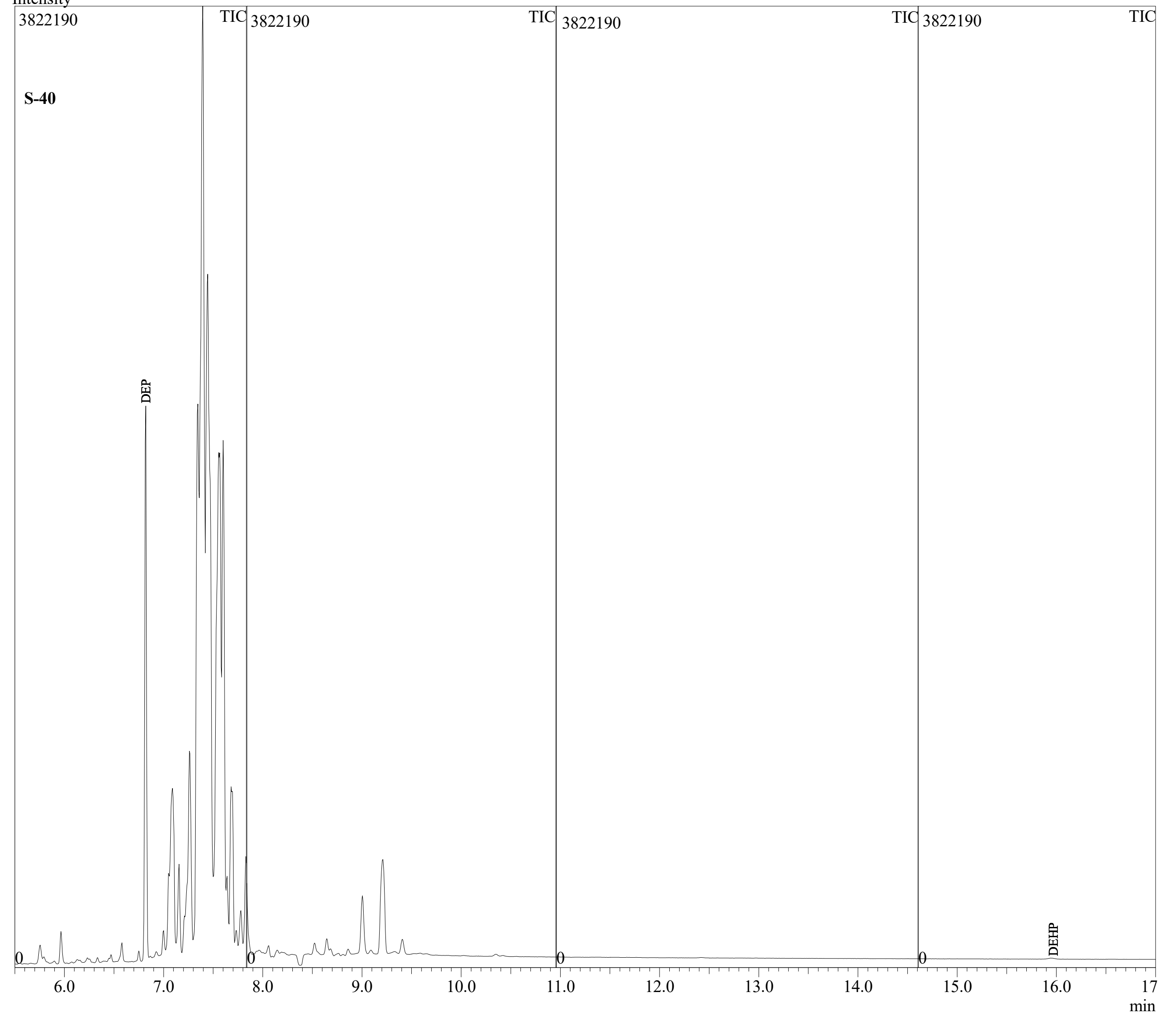
Intensity
3822190
TIC
S-40
DEP
DEHP
0
6.0
7.0
8.0
9.0
10.0
11.0
12.0
13.0
14.0
15.0
16.0
17
min
